# Mfsd2a-mediated lysolipid transport is important for renal recovery after acute kidney injury

**DOI:** 10.1016/j.jlr.2023.100416

**Published:** 2023-07-17

**Authors:** Randy Y.J. Loke, Cheen Fei Chin, Gao Liang, Bernice H. Wong, Dwight L.A. Galam, Bryan C. Tan, Geok-Lin Chua, Shintaro Minegishi, Norihiko Morisawa, Iulia Sidorov, Bram Heijs, Jens Titze, Markus R. Wenk, Federico Torta, David L. Silver

**Affiliations:** 1 Signature Research Program in Cardiovascular and Metabolic Disorders, Duke-National University of Singapore (NUS) Medical School, Singapore; 2 Singapore Lipidomics Incubator, Life Sciences Institute, NUS, Singapore; 3 Department of Biochemistry, Yong Loo Lin School of Medicine, NUS, Singapore; 4 Center of Proteomics and Metabolomics, Leiden University Medical Center, Leiden, the Netherlands; 5 The Novo Nordisk Foundation Center for Stem Cell Medicine (reNEW), Leiden University Medical Center, Leiden, the Netherlands

**Keywords:** Acute kidney injury, Phospholipid, Transporter, Mfsd2a, omega-3 fatty acid, DHA

## Abstract

Acute kidney injury (AKI) is a global public health concern with high mortality and morbidity. In ischemic–reperfusion injury (IRI), a main cause of AKI, the brush border membrane of S3 proximal tubules (PT) is lost to the tubular lumen. How injured tubules reconstitute lost membrane lipids during renal recovery is not known. Here, we identified Mfsd2a, a sodium-dependent lysophosphatidylcholine (LPC) transporter, to be expressed specifically in the basolateral membrane of S3 PT. Using an in vivo activity probe for Mfsd2a, transport activity was found to be specific to the S3 PT. Mice with haploinsufficiency of Mfsd2a exhibited delayed recovery of renal function after acute IRI, with depressed urine osmolality and elevated levels of histological markers of damage, fibrosis, and inflammation, findings corroborated by transcriptomic analysis. Lipidomics revealed a deficiency in docosahexaenoic acid (DHA) containing phospholipids in Mfsd2a haploinsufficiency. Treatment of Mfsd2a haploinsufficient mice with LPC-DHA improved renal function and reduced markers of injury, fibrosis, and inflammation. Additionally, LPC-DHA treatment restored S3 brush border membrane architecture and normalized DHA-containing phospholipid content. These findings indicate that Mfsd2a-mediated transport of LPC-DHA is limiting for renal recovery after AKI and suggest that LPC-DHA could be a promising dietary supplement for improving recovery following AKI.

AKI continues to be a global public health concern, impacting an estimated 13.3 million patients per year (1). The global burden of AKI-related mortality is high at 20%–50% depending on the economic status of the country and the stage of AKI. In developed countries, AKI mainly occurs in hospital settings and is especially common in patients in intensive-care units, with a prevalence of up to 60% (2). Despite the high prevalence of AKI, current treatments are supportive as no targeted therapies exist (1). AKI has also been shown to contribute to the development of end-stage renal disease requiring dialysis. A deeper understanding of the pathophysiology of AKI will be important in developing new therapies for the treatment of AKI.

Ischemic–reperfusion injury (IRI) is one of the main causes of AKI. In recent years, there have been many advances in understanding the pathological mechanisms of AKI from animal models of IRI. There are multiple pathologic processes following IRI, including endothelial dysfunction, vasoconstriction of regional circulation, generation of reactive oxygen species, tubular injury, production of inflammatory cytokines, and recruitment and cross-talk with immune cells (3). Irreversible renal damage and fibrosis are the long-term sequelae that contributes to chronic kidney disease.

The S3 segment of the PT is most susceptible to ischemia in both animal models and human patients (4). Thus, the S3 segment is generally regarded as an important anatomical site in the kidney for understanding the pathophysiology of AKI. Human biopsies of AKI samples have revealed S3 segment-specific detachment of tubular cells from basement membranes, sloughing of the epithelium into the luminal space, effacement and loss of brush border membrane, and formation of tubular casts from cell debris and sloughed membranes (5).

Phosphoglycerolipids, sphingolipids, and cholesterol play important roles in membrane bilayer structure, cell signaling, and in the functioning of membrane proteins (6). Multiple studies demonstrated that AKI induces membrane instability through lipid dysregulation (7, 8). These changes may be either protective or cyotoxic (9). For instance, increases in cholesterol content in plasma membranes following ischemia is believed to confer protection against kidney damage, a term known as “cytoresistance”. Plasmalogens such as *sn1* ether-linked phosphatidylcholine (PC-P) and phosphatidylethanolamine (PE-P) are enriched in proximal tubules of the kidney where they are thought to modulate membrane fluidity and may have antioxidant properties (10). However, ether lipids can also be hydrolyzed by phospholipase A2 enzymes during IRI, which leads to the accumulation of free fatty acids such as arachidonic acid, a precursor of inflammatory molecules including prostaglandins, leukotrienes, and thromboxanes (11).

The brush border membrane of the S3 segment is structurally unique with a thick, highly developed microvilli surface not seen in S1 and S2 segments of the PT. The brush border and basolateral membranes of PT contain high amounts of cholesterol among neutral lipids species, while sphingomyelin, PC, and PE are the main species in the phospholipid pool (12). Single-cell sequencing has also revealed the expression of enzymes in the Kennedy pathway for the biosynthesis of phosphatidylcholine and enzymes for the de novo synthesis of polyunsaturated fatty acids (13). However, it remains unknown how specific segments of the kidney, such as S3, acquire their unique set of membrane lipids.

We originally identified Major Facilitator Superfamily Domain-containing 2a (Mfsd2a) as a sodium-dependent LPC transporter highly expressed in the endothelium of the blood–brain barrier and blood–retinal barrier (14, 15). Mfsd2a is the primary transporter for the uptake of omega-3 fatty acid DHA (LPC-DHA) in the brain and eye. Mfsd2a exhibits specificity for LPC species containing mono- and polyunsaturated fatty acids (14). Recent cryo-EM structures raised the possibility that Mfsd2a transports LPC by a flippase-type mechanism (16, 17, 18, 19). Indeed, reconstitution of Mfsd2a transport activity in proteoliposomes demonstrated that Mfsd2a functions as an LPC flippase (20). The physiological importance of Mfsd2a in brain development and function is underscored by the observation that Mfsd2a knockout mice (2aKO) display severe microcephaly, hypomyelination deficiency in brain DHA, and deficits in learning and memory (14, 21). Moreover, individuals with loss-of-function mutations in MFSD2A (a.k.a Microcephaly 15 Autosomal Recessive) present with severe microcephaly and hypomyelination, indicating that LPC transport via Mfsd2a is essential for normal human brain development and myelination (22, 23, 24, 25). Microcephaly in Mfsd2a deficiency mouse models was shown to be due to decreased neuron arborization and not due to neuronal cell loss (26). Additionally, LPC-DHA transported by Mfsd2a negatively regulated SREBP1 processing such that Mfsd2a deficiency resulted in significant changes in phospholipid membrane saturation due to upregulation of SREBP1 activity (26). These findings indicate that LPCs transported by Mfsd2a act as membrane phospholipid precursors for building membranes as well as regulating membrane composition through control of SREBP1 processing.

Mfsd2a is not exclusively expressed in brain and eye blood barriers but also in liver, alveolar type II cells in the lung, and kidney (27, 28). The role of Mfsd2a in the kidney remains unknown but raises the possibility that LPCs transported by Mfsd2a in the kidney are utilized to replace damaged membrane phospholipids, a process that might become limiting post-injury.

## Materials and Methods

### Animals

All mice were bred and maintained at the Duke-NUS animal facility. All experimental protocols are approved by Singhealth Institutional Animal Care and Use Committee (IACUC protocol number #2015/SHS/1416). Mfsd2a heterozygous (HET) and Mfsd2a knockout (2aKO) mice were generated as described previously (26). All mice were housed in cages on a 12-h light/12-h dark cycle with controlled humidity and temperature at 23°C. Animals were fed *ad libitum* on a normal chow diet (12% fats, 23% protein, and 65% carbohydrates) and had free access to water. Male mice 8–10 weeks of age were selected for animal experiments.

Mfsd2a-CreERT2 Rosa26-tdtomato mice were generated by crossing Mfsd2a-specific tamoxifen-inducible Cre to ROSA26-tdTomato reporter mice (29). To induce recombination, tamoxifen was given by intraperitoneal injection of 100 μg/g bodyweight of the mouse for 4 consecutive days.

Tamoxifen-dependent, doxycycline-inducible Mfsd2a overexpression mouse line (iMfsd2a mice) was generated by crossing of Rosa26-Mfsd2a mice with Mfsd2a-CreERT2 Rosa26-tdtomato mice (supplemental Fig. S1). With tamoxifen treatment, Cre-ERT2 excises loxP sites at Rosa26 locus allowing rtTA expression. rtTA drives the expression of Mfsd2a in the presence of doxycycline.

### Tamoxifen/doxycycline treatment

One mg tamoxifen (Thermo Fisher Scientific, Cat# T5648) was dissolved in 10 ml absolute ethanol and suspended in 50 ml of corn oil. The 20 mg/ml tamoxifen solution was sonicated for 2 min and ethanol was blow dry with a nitrogen stream. Tamoxifen was given by the intraperitoneal injection of 100 μg/g bodyweight of mouse for four days.

100 mg doxycycline (Sigma, Cat# 9891) was dissolved in 10 ml sterilized PBS to make a stock solution of 10 mg/ml. Doxycycline is given via intraperitoneal (i.p.) route at a dosage of 50 μg/g bodyweight of mouse.

### IRI procedure

A nephrectomy of the right kidney was performed when the mice were 8–10 weeks of age. Animals were anesthetized with an intraperitoneal injection of ketamine/xylazine (100/10 mg/kg). A small incision through the right flank muscle and fascia above the kidney was made to exteriorize the right kidney. The renal pedicle is ligated and cut distal to the suture. The kidney was removed, and the peritoneum and skin closed with 4/0 silkam sutures.

One week after the right nephrectomy, ischemic kidney injury procedure was performed. A small incision was made through the left flank muscle and fascia above the kidney to exteriorize the left kidney. A non-traumatic microvascular clamp (Roboz Surgical Instrument) was placed on the renal pedicle for 55 min. The 55 min duration was chosen with a titration test where tissue injury, casts, fibrosis, and repair were consistently seen with 55-min clamping at postoperative day 10. After the removal of clamps, wounds were closed with 4/0 silkam sutures and animals were allowed to recover. During the procedure, animals were kept warm with a heat lamp until awake. The mice were then placed into metabolic cages for 10 days, with daily measurements of urine output, water intake, and bodyweight. Mice were sacrificed and kidneys harvested for histology, lipidomic, and transcriptomic analysis.

### LPC-DHA treatment

After IRI, the mice were randomly divided into 2 groups (n ≥6 per group): (i) a control group, in which mice were i.v injected once daily with 12% BSA in saline for 10 days and (ii) a LPC-DHA group, in which mice were i.v injected once daily with 8 mg/kg bodyweight LPC-DHA (2 mg/ml solution in sterile normal saline 12% BSA, a kind gift from Travecta Therapeutics). Mice were housed in metabolic cages for 10 days with daily measurements of urine output, water intake, and bodyweight. Mice were sacrificed and kidneys were harvested for histology, lipidomic, and transcriptomic analysis.

### Urine collection

The mice were placed into metabolic cages (CLEA Japan, Inc./CL-0355) 2 days before ischemic surgery to acclimatize to the metabolic cages with free access to regular chow diet and water. The 24 h urine volume and fluid intake were measured daily for 10 days.

### Osmolality measurement

Plasma and urine osmolality were measured by vapor pressure osmometry (VAPRO®).

### Histology

Kidneys were dissected and fixed in 4% paraformaldehyde (ICM Pharma) for 24 h followed by 70% ethanol, paraffin-embedded, and cut to 5 μm thickness. The sections were then stained with PAS, and acid-fast green and subjected to immunohistochemistry (IHC). For IHC, paraffin-embedded kidney sections were dewaxed, rehydrated, and antigen retrieval was carried out in microwave-boiled citrate buffer (10 mM citric acid, pH 6.0) for 1 h (KIM-1) or 10 min in Protenase K solution (20 μg/ml Proteinase K, 50 mM Tris, 1 mM EDTA, pH 8.0). Slides were then incubated with BLOXALL blocking solution (Vector laboratories) for 30 min and washed 3 times with TBS-T (50 mM Tris-HCl pH7.40, 150 mM NaCl, 0.1% Tween-20). Blocking was done with blocking buffer containing 2.5% horse serum in TBS-T (for anti-KIM-1) or 5% Normal goat serum in TBS-T (for anti-F4/80) for 1 h and incubated overnight at 4°C with primary antibody diluted in respective blocking buffers. Anti-KIM-1 (R&D systems) was used at 1:800 dilution and Anti-F4/80 (BioRad) was used at 1:400 dilution. After overnight incubation, sections were rinsed 3 times with TBS-T and incubated with horseradish peroxidase–conjugated secondary antibody (Vector Laboratories) for 1 h. Sections were rinsed 3 times with TBS-T and then subjected to 3, 3′-Diaminobenzidine (Vector Laboratories) staining. Hematoxylin counterstain of nuclei was done before mounting with CV ultra-mounting media (Leica Biosystems). Images were captured using a light microscope (Leica) with the 20x objective.

### PAS staining

Deparaffined sections were stained for PAS using PAS-Staining Kit (Sigma-Aldrich). In brief, the slides were treated for 5 min in periodic acid solution, rinsed twice with distilled water, 15 min in Schiff’s reagent, 2 rinses, 15 min in Meyer’s hematoxylin (Vector laboratories) solution, 2 rinses, and serial dehydration in 70%, 80%, 90%, 100% ethanol before putting on the coverslip.

### Fast green staining

Deparaffined sections were stained with fast green (FCF, Sigma-Aldrich) for 5 min, rinsed, and then dehydrated and cleared with absolute ethanol and xylene for 2 min each.

### Histology quantification

IHC stained area was quantified with ImageJ and represented as % area of whole kidney section to avoid bias in choosing image fields.

### Immunofluorescence

Paraffin-embedded kidney sections were dewaxed, rehydrated, and antigen retrieval was carried out in microwave-boiled citrate buffer (10 mM citric acid, pH 6.0) for 1 h (fibronectin and Ki-67 staining). Sections were then permeabilized with 0.1% Triton X-100 in PBS (137 mM NaCl, 2.7 mM KCl, 10 mM Na2HPO4, 1.8 mM KH2PO4) and blocked with blocking buffer [5% Normal goat serum, 0.1% Triton X-100 in PBS (PBS-TX)] for 1 h. Incubation with primary antibody diluted in blocking buffer is done at 4°C overnight. Anti-fibronectin (Abcam) at 1:250 dilution, anti-Ki-67 (Abcam) at 1:500 dilution, anti-Mfsd2a (in house) at 1:100, anti-RFP (Rockland) at 1:500 dilution was used. Sections are then washed three times the following day with PBS-TX and incubated with Alexa Fluor Plus secondary antibody (Thermo Fisher Scientific) diluted in blocking buffer (1:250) for 1 h. After washing three times with PBS-TX, Hoechst 33,342 (Thermo Fisher Scientific) was used to stain nuclei and the slides mounted with Fluorsave Reagent (Merck). Leica Fluorescence microscope (Leica Biosystems) was then used for image acquisition.

### *In vitro* and *in vivo* transport assays

*In vitro* transport of LPC-Lightox was carried out on HEK293 cells harboring a Dox-inducible wild-type Mfsd2a and transport dead mutant Mfsd2a-D97A construct respectively. To determine if LPC-LightOx can be transported into the epithelium of the S3 segment, LPC-LightOx was injected into WT and Mfsd2a whole-body knockout mice (2aKO) and 2 h later kidneys were perfused, fixed, and sectioned.

### RNA isolation

Total RNA extraction from the kidney was performed using Trizol according to standard protocol. Briefly, the kidney was homogenized in Trizol with MagNALyser (Roche) and purified with RNeasy Mini kit (Qiagen). RNA concentration was measured using NanoDrop Spectrophotometer (Thermo Fisher Scientific).

### Quantitative real-time reverse-transcription PCR

Kidney cDNA was synthesized from 1 μg total RNA with iScriptTM Reverse Transcriptase Supermix (Bio-Rad) as per manufacturer instructions. SensiFAST^TM^SYBR® Hi-ROX Kit (Bioline) was used for qPCR and Mfsd2a mRNA normalized to β-actin as a housekeeping gene.

### RNA-sequencing

Kidney RNA library preparation and RNA-sequencing were performed by Novogene AIT (Singapore). RNA-Seq analysis was performed with Partek Flow (version 10). The RNA-seq reads were mapped against mm10 using STAR 2.5.4 b aligner and the raw counts were calculated with FeatureCounts. Features were filtered using recommended parameters and median ratio was normalized. Differential expression analysis was performed using DESeq2, and a gene was considered significantly differentially expressed when *P*-value <0.05 and fold change ≥ 2.0.

### Sample preparation for lipidomic analysis

10 μl of homogenized kidney in 150 mM ammonium bicarbonate (1:20, v/v) were mixed with 490 μl of butanol:methanol (1:1, v/v) spiked with deuterated internal standards (IS). The standards used were acylcarnitine 16:0 D3, cholesterol ester 18:0 D6, ceramide d18:0/08:0, ceramide d18:1/12:0, deoxyceramide m18:1/12:0, cholesterol D7, diacylglycerol 15:0/15:0, GM3 ganglioside d18:1/18:0 D3, monohexosylceramide d18:1/12:0, dihexosylceramide d18:1/12:0, trihexosylceramide d18:1/18:0 D3, lysophosphatidylcholine 13:0, lysophosphatidylethanolamine 14:0, phosphatidylcholine 13:0/13:0, plasmalogen phosphatidylcholine 18:0/18:1 D9, phosphatidylethanolamine 17:0/17:0, plasmalogen phosphatidylethanolamine 18:0/18:1 D9, phosphatidylglycerol 17:0/17:0, phosphatidylinositol 12:0/13:0, phosphatidylserine 17:0/17:0, sphingomyelin d18:1/12:0, sphinganine d17:0, sphingosine d17:1, triacylglycerol 12:0/12:0/12:0 and were purchased from Avanti Lipids. The mixture was vortexed for 2 min, sonicated for 30 min, and then centrifuged twice at 4°C (14,000 g for 10 min). The supernatant fraction was collected for LC-MS/MS analysis. A pooled lipid extract was used as a quality control (QC) sample and injected every 5 study samples.

### LC-MS/MS analysis

The LC-MS/MS analysis was performed on an Agilent UHPLC 1290 Infinity II liquid chromatography system connected to an Agilent QqQ 6495C.

#### LC

An Agilent ZorbaxRRHD Eclipse Plus C18 column (2.1 × 50 mm, 1.8 μm) was used for the RPLC separation. The mobile phases A (60% water and 40% acetonitrile with 10 mmol/L ammonium formate) and B (10% acetonitrile and 90% isopropanol with 10 mmol/L ammonium formate) were used for the chromatographic separation. The following gradient was applied: 0–2 min, 20%–60% B; 2–12 min, 60%–100% B; 12–14 min, 100% B; 14.01–15.8 min, 20% B. The oven temperature was maintained at 40°C. The flow rate was set at 0.4 ml/min and the sample injection volume was 1 μl.

#### QqQ

The positive ionization spray voltage and nozzle voltage were set at 3000 V and 1000 V, respectively. The drying gas and sheath gas temperatures were both maintained at 250°C. The drying gas and sheath gas flow rates were 14 L/min and 11 L/min, respectively. The nebulizer nitrogen gas flow rate was set at 35 psi. The iFunnel high- and low-pressure RF were 150 V and 60 V, respectively. Targeted analysis was performed in Dynamic MRM positive ion mode.

#### Data analysis

The acquired MS data were analyzed using Agilent MassHunter software version B.10.00.

#### Normalization and quantification

The signal-to-noise ratios (S/N) were calculated using the raw peak areas in study samples and processed blanks (PBLK). Lipids that had S/N < 10, CV > 20% in the QC samples and did not show a linear behavior (*R*^2^< 0.8) in dilution curves were excluded from the analysis. Internal standards were used to normalize the raw peak areas in the corresponding lipid class, and concentrations were further normalized to the protein concentration in the original sample. Due to the methodology used in our study, we are not able to report absolute concentration values. Endogenous species were quantified using one standard per lipid class thus our method can only deliver relative quantitation results.

### Quantification and statistical analysis

Samples were biological replicates, represented as mean ± standard deviation (S.D). Two-tailed unpaired Welch’s *t* test was used to analyze differences between experimental groups. *P* values less than 0.05 were considered statistically significant. The analysis was performed using Prism 9 (GraphPad Software).

### Graphical illustrations

Graphical illustrations were generated with Biorender.com. Graphs in the figure were created with GraphPad Prism 9.

## Results

### Mfsd2a transports LPC specifically in the S3 proximal tubules of the kidney

**Fig. 1:**
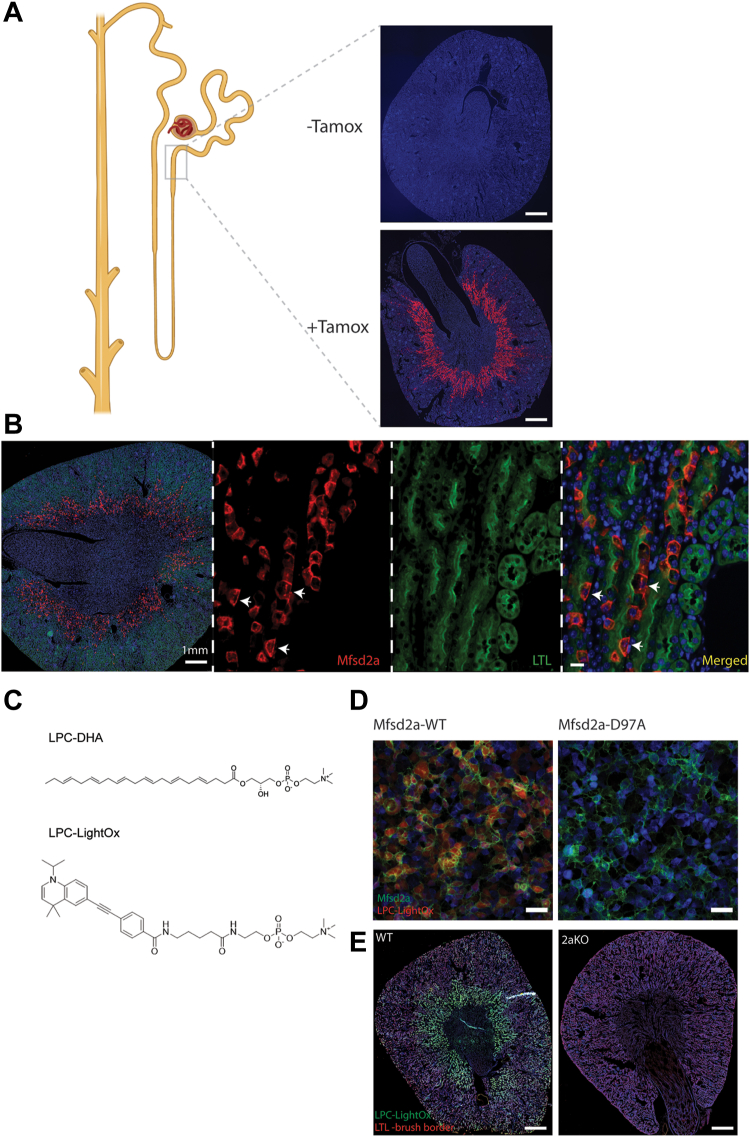
Mfsd2a is expressed in S3 proximal tubules and transports LPCs in vivo. A: localization of Mfsd2a expression by lineage tracing using Mfsd2aKiCre-TdTomato mice. A model of the nephron shown on the left indicating the location of the Mfsd2a expression in the PT. Tamoxifen treatment of Mfsd2aKiCre-TdTomato mice shows TdTomato expression at the S3 segment (corticomedullary junction). No tamoxifen treatment served as negative control (top panel). n = 3 mice per genotype. Scale bar = 1.5 mm. B: Low-magnification image on the leftmost panel showing S3-specific expression of TdTomato. The following three higher magnification sections were labeled with Mfsd2a (red), Hoechst (blue), and LTL (green), a marker of proximal tubules. The staining pattern indicates the basolateral expression of Mfsd2a (white arrows). Scale bar = 50 μm. C: Structure of LPC-DHA and LPC-LightOx. D: in vitro uptake of LPC-LightOx in HEK293 cells with doxycycline-inducible Mfsd2a-GFP expression. LPC-LightOx is taken up by cells expressing Mfsd2a-GFP. Mfsd2a-D97A-GFP is a transport dead mutant and served as a negative control. The experiment was performed with three technical replicates. Scale bar = 50 μm. E: LPC-LightOx (green) was i.v. injected into WT and 2aKO mice and kidneys were collected 2 h later. A section through a WT kidney shows LPC-LightOx uptake into the S3 segment but is absent from 2aKO (n = 3 mice per genotype). Scale bar = 1.5 mm.

We originally demonstrated Mfsd2a protein to be expressed in mouse kidney, but the cell types and anatomical location of Mfsd2a expression in the kidney were not known (27). Utilizing a recently developed single-cell transcriptomics database from a mouse kidney, we found Mfsd2a to be exclusively expressed in the S3 segment of the PT (13). To confirm this finding, we utilized a tamoxifen-inducible lineage tracing line we generated for Mfsd2a that faithfully reports on the expression of Mfsd2a at the blood-brain barrier (Mfsd2aKiCre-TdTomato) (30). Following tamoxifen treatment of Mfsd2aKiCre-TdTomato mice, we found TdTomato to be expressed exclusively in S3 proximal tubules (Fig. 1A). These findings indicate that Mfsd2a expression is specific for epithelial cells of the S3 segment.

Proximal tubules play a major role in the reabsorption of solutes from the glomerular filtrate and are characterized by morphological and functional polarity of membranes and transporters surrounding the lumen of the tubules on one side (apical) and the interstitium on the other side (basolateral). The luminal space of the proximal tubule has a high concentration of sodium (140–150 mmol/L) (31, 32) relative to intracellular sodium concentration (10–15 mmol/L), within the range to drive sodium-dependent LPC transport by Mfsd2a (14, 20). The location of Mfsd2a to either the apical or the basolateral membranes in epithelial cells of the S3 will have important implications on its function. The apical expression would indicate a function in the recycling of LPC in the glomerular filtrate while basolateral expression would indicate the uptake of LPC from the blood into the epithelial cell.

To determine the membrane localization of Mfsd2a we first sought to use our in-house Mfsd2a antibody, but it was unable to detect Mfsd2a in kidney tissues, possibly due to epitope masking or inadequate antibody affinity. To circumvent this issue, we generated a tamoxifen-dependent, doxycycline-inducible Mfsd2a overexpression mouse line we termed iMfsd2a (supplemental Fig. S1A, B). We crossed iMfsd2a mice to our lineage tracing line Mfsd2aKiCre to induce overexpression specifically under the control of the Mfsd2a locus. Immunofluorescence microscopy performed on kidney sections from these mice revealed that Mfsd2a localized specifically at basolateral membranes in epithelial cells of the S3 segment (Fig. 1B). It is notable that not all epithelial cells in the S3 segment express Mfsd2a in the iMfsd2a line, which is not observed for Mfsd2aKiCre-TdTomato (Fig. 1A). This finding suggests that the cause of chimeric expression of Mfsd2a is due to the iMfsd2a transgene rather than the Mfsd2aKiCre transgene. Nonetheless, these data suggest that Mfsd2a transports LPC from nearby blood vessels into epithelial cells of the S3 segment. We sought to directly test this hypothesis by demonstrating LPC transport into the S3 segment. However, a probe to study Mfsd2a transport activity in vivo that can give spatial resolution in complex tissues like the kidney has not previously been developed. The current state-of-the-art is the use of LPC labeled with fluorophores such as NBD that emit in the green wavelength (14). These probes have proven problematic due to tissue autofluorescence and poor in vivo stability. To overcome these limitations, we set out to design and synthesize a Mfsd2a activity probe that has high Mfsd2a specificity and in vivo stability. In brief, we chose LightOx as the fluorophore because it is UV activated and emits in the blue wavelengths in which tissues have very low background fluorescence (33). In addition, we modified the LPC backbone to prevent in vivo catabolism/metabolism by endogenous phospholipases and acyltransferases. We named this probe LPC-LightOx (Fig. 1C). Importantly, LPC-LightOx was taken up by cells in a Mfsd2a-dependent fashion (Fig. 1D). To determine if LPC-LightOx can be taken up into the epithelium of the S3 segment in a Mfsd2a-dependent fashion, LPC-LightOx was injected into WT and 2aKO mice and 2 h later kidneys were perfused, fixed and sectioned. Scanning of kidney sections revealed that LPC-LightOx was taken up with remarkable specificity into epithelial cells of the S3 segment in a Mfsd2a-dependent fashion (Fig. 1E). These data indicate that blood-derived LPC is specifically transported into the epithelium of the S3 segment by Mfsd2a expressed at the basolateral membrane of these cells.

### Haploinsufficiency of Mfsd2a negatively affects renal function and recovery after IRI

**Fig. 2:**
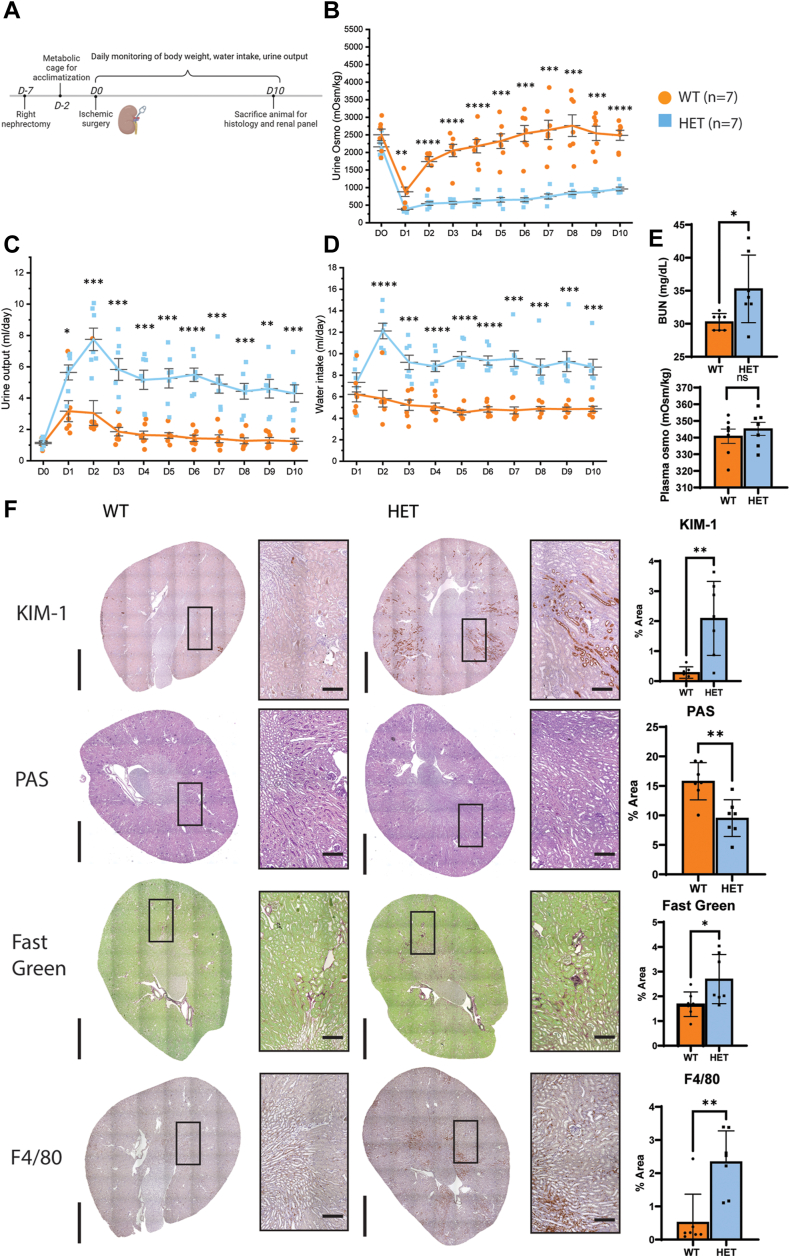
HET mice have impaired renal function in response to ischemic injury. A: an experimental timeline for ischemic surgery and metabolic cage monitoring. B: urine osmolality over the course of 10 days after IRI surgery. C: urine output, and (D) water intake measurements per day. E: BUN levels at day 10. Data are represented as mean ± S.D. F: histological staining of markers of kidney injury (KIM-1), fibrosis (fast green), and inflammation (F4/80) between WT and HET animals at day 10. Representative images are shown. Marker levels were quantified as % area of total kidney area and represented as mean ± SD. n = 7 WT, 7 HET mice. Scale bar = 3 mm and 100 μm, respectively. *p* < 0.0001 ∗∗∗∗, *P* < 0.001 ∗∗∗, *P* < 0.01 ∗∗, *P* < 0.05 ∗.

Given that the S3 segment is the most sensitive part of the PT and generally across the kidney to renal ischemic damage and Mfsd2a expression is specific to this segment of the PT, we wanted to study the role of LPC transport by Mfsd2a in the context of renal ischemia using Mfsd2a deficiency mouse models. The use of 2aKO mice is potentially problematic for studying renal function because they present with several major pathologies and phenotypes such as small size and leanness, microcephaly and behavioral abnormalities, and changes in whole-body energy expenditure and food intake relative to wild-type (WT) controls (14, 27). Because of these confounding factors, we chose to focus our studies on mice heterozygous for deletion of *Mfsd2a* (HET) that express 50% of the transcript levels of Mfsd2a in the kidney relative to WT mice (supplemental Fig. S2). Our experimental protocol involves a unilateral IRI setup (Fig. 2A) by first performing a right nephrectomy on groups of WT and HET mice and allowing the mice to recover for five days before acclimating them to metabolic cages for two days. Mice then undergo renal artery clamping and reperfusion followed by daily monitoring of body weight, water intake, and urine collection for downstream analyses. The advantage of this approach over measurements of plasma creatine alone is that this protocol allows for the quantification of urine osmolality, a direct measurement of the concentrating function of the PT. The rationale for removing one kidney prior to inducing IRI is to allow for an assessment of the function of the damaged kidney which would be masked by the presence of a healthy kidney. Prior to inducing IRI, both WT and HET mice had similar urine osmolality, urine output, and water intake (Fig. 2B–D). At one day post-IRI, both WT and HET mice showed a similar decline in urinary osmolality and increased urine output and water intake. Histological analysis on day 2 post-IRI revealed very similar levels of kidney injury in WT and HET mice (supplemental Fig. S3). While WT mice exhibited prompt recovery of urine osmolality after IRI with osmolality returning to baseline by day 4 and 5, HET animals continued to exhibit low urine osmolality 10 days after IRI injury, indicating impairment of kidneys to concentrate urine. Consistent with reduced kidney function, HET mice also exhibited polyuria and polydipsia (Fig. 2C, D), common compensatory mechanisms for low urine osmolality. Plasma osmolality, however, was unchanged between WT and HET mice, indicating normal hypothalamic function to regulate plasma osmolality (Fig. 2E) with only a moderate elevation in BUN (Fig. 2E) indicating minor effects on the glomerulus. Taken together, these data indicate haploinsufficiency of Mfsd2a negatively impacted recovery of kidney function following IRI.

To better understand the renal pathology in HET mice following IRI we performed a series of histological analyses at day 10 post-IRI. Day 10 was chosen because this is well beyond the stage at which WT mice showed full recovery of renal function while HET mice did not (Fig. 2B–D). Prior to IRI, kidneys of HET mice did not have noticeable pathologies and were similar to kidneys from WT mice in terms of S3 brush border thickness (PAS staining), lack of inflammatory cells (F4/80), lack of fibrosis (fast green), and absence of KIM-1, an established marker of kidney injury (supplemental Fig. S4) (34). These findings are consistent with normal renal function in HET prior to IRI (Fig. 2). In contrast, at day 10 post-IRI HET kidneys exhibited significantly higher KIM-1 staining in the S3 segment than WT kidneys (Fig. 2F). PAS staining revealed more areas of denuded tubules, casts as well as thinner brush borders of the S3 segment in HET relative to controls that appeared to have completely recovered by day 10. HET kidneys also exhibited significantly more collagen and fibronectin deposition and macrophage infiltration than WT controls (Fig. 2F and supplemental Fig. S6), indicative of kidney fibrosis and inflammation. Kidney injury often results in more tubule epithelial cells undergoing proliferation as part of the remodeling and repair process (35). Indeed, Ki-67 staining revealed significantly increased levels of mitotic epithelial cells in the HET kidneys relative to WT kidneys consistent with kidney injury (supplemental Fig. S6).

**Fig. 3:**
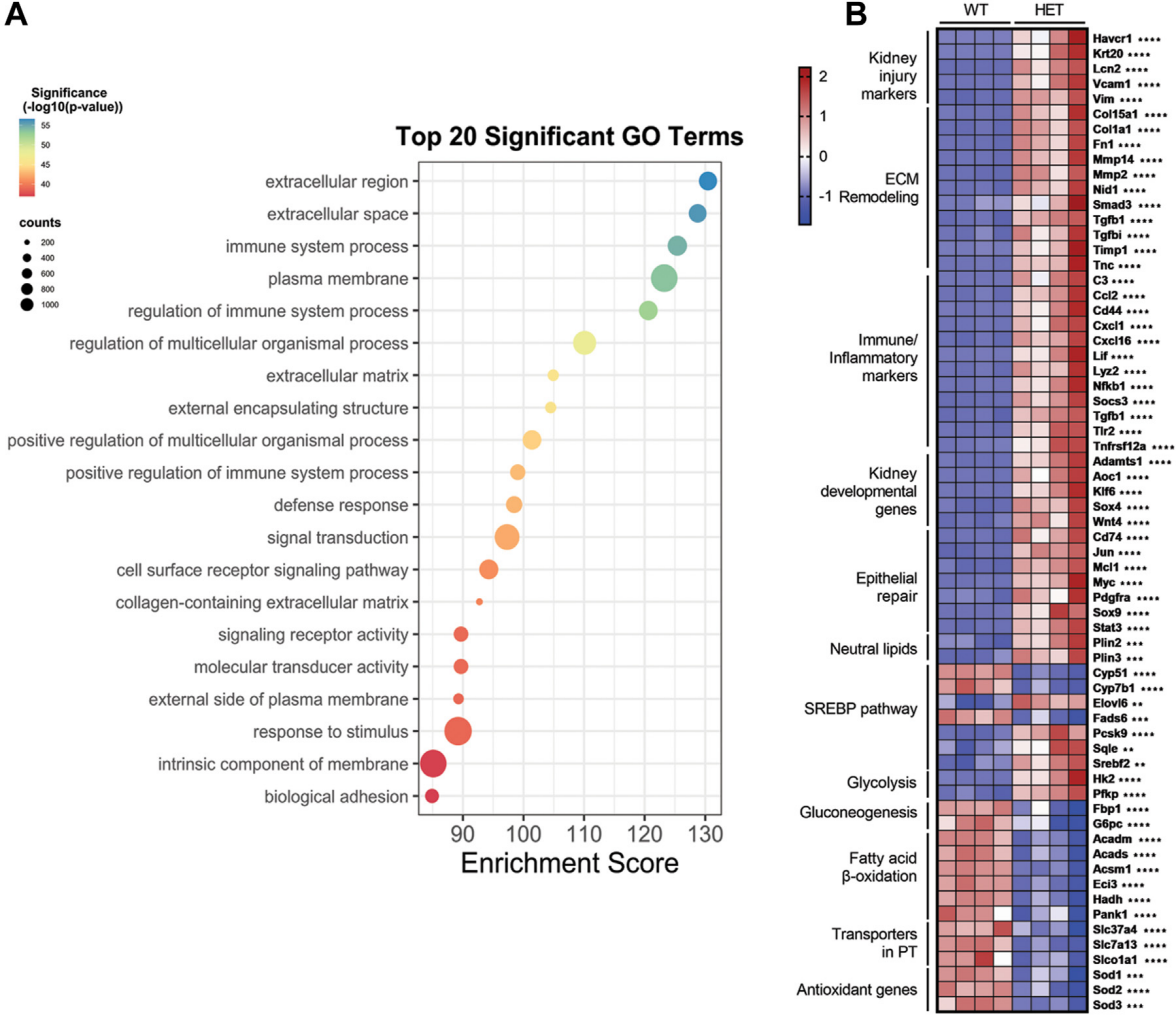
Transcriptomic changes indicative of poor renal recovery in HET kidneys. A: Transcriptomic analysis of kidney tissues in WT and HET 10 days after surgery. Top 20 Gene Ontology (GO) pathways upregulated in the HET relative to WT kidneys shown. B: Heatmap with indicated genes grouped into pathways that are significantly different between HET and WT mice. n = 7 WT, 7 HET mice. *P* < 0.0001 ∗∗∗∗, *P* < 0.001 ∗∗∗, *P* < 0.01 ∗∗, *P* < 0.05 ∗.

To more deeply understand the consequences of Mfsd2a haploinsufficiency on kidneys post-IRI, we performed a bulk RNA-Seq analysis of kidneys from WT and HET mice 10 days post-IRI. We identified a total of 5149 significantly upregulated and 1010 significantly downregulated differentially expressed genes. Gene ontology analysis revealed that among the most significantly upregulated pathways in the HET kidneys were inflammation and fibrosis (Fig. 3A). Within the fibrosis pathway, genes central to fibrogenesis such as collagen, matrix metalloproteinases, and *Tgf-β* were significantly upregulated. We additionally identified a corresponding increase in many immune-inflammatory markers in HET kidneys such as toll-like receptors (*Tlr2, Tlr4, Tlr7*), *Stat1*, *Stat2*, and chemokines (*Cxcl1, Ccl2, Cxcl16*) (Fig. 3B). These gene expression changes corroborated the histopathological findings of increased fibrosis and inflammatory cells in HET mice (Fig. 2F). Biomarkers of kidney injury such as Havcr1 (aka KIM-1 also seen histologically, Fig. 2F), *Lcn2*, *Vcam1* were also upregulated in HET kidneys (Fig. 3). Energy metabolism pathways were also dysregulated in HET kidneys. Enzymes involved in fatty acid beta-oxidation (*Acads, Acadm, Acadl, Acadvl, Hadh, Acaa2, Echs1*) were significantly decreased while those in glycolysis (*Hk2, Pfkp, Pklr*) were upregulated in kidneys of HET mice. These data indicate that the injured kidneys of HET mice had altered energy substrate utilization with a shift from fatty acid oxidation to glycolysis.

In summary, these data indicate that WT kidneys made a full recovery by day 10 post-IRI while HET mice presented with histopathological findings and kidney dysfunction consistent with severe renal injury. These findings suggest that Mfsd2a is limiting for kidney repair after AKI. We next sought to understand the underlying mechanism for these findings.

### Haploinsufficiency of Mfsd2a results in reduced DHA-containing phospholipids post-IRI

**Fig. 4:**
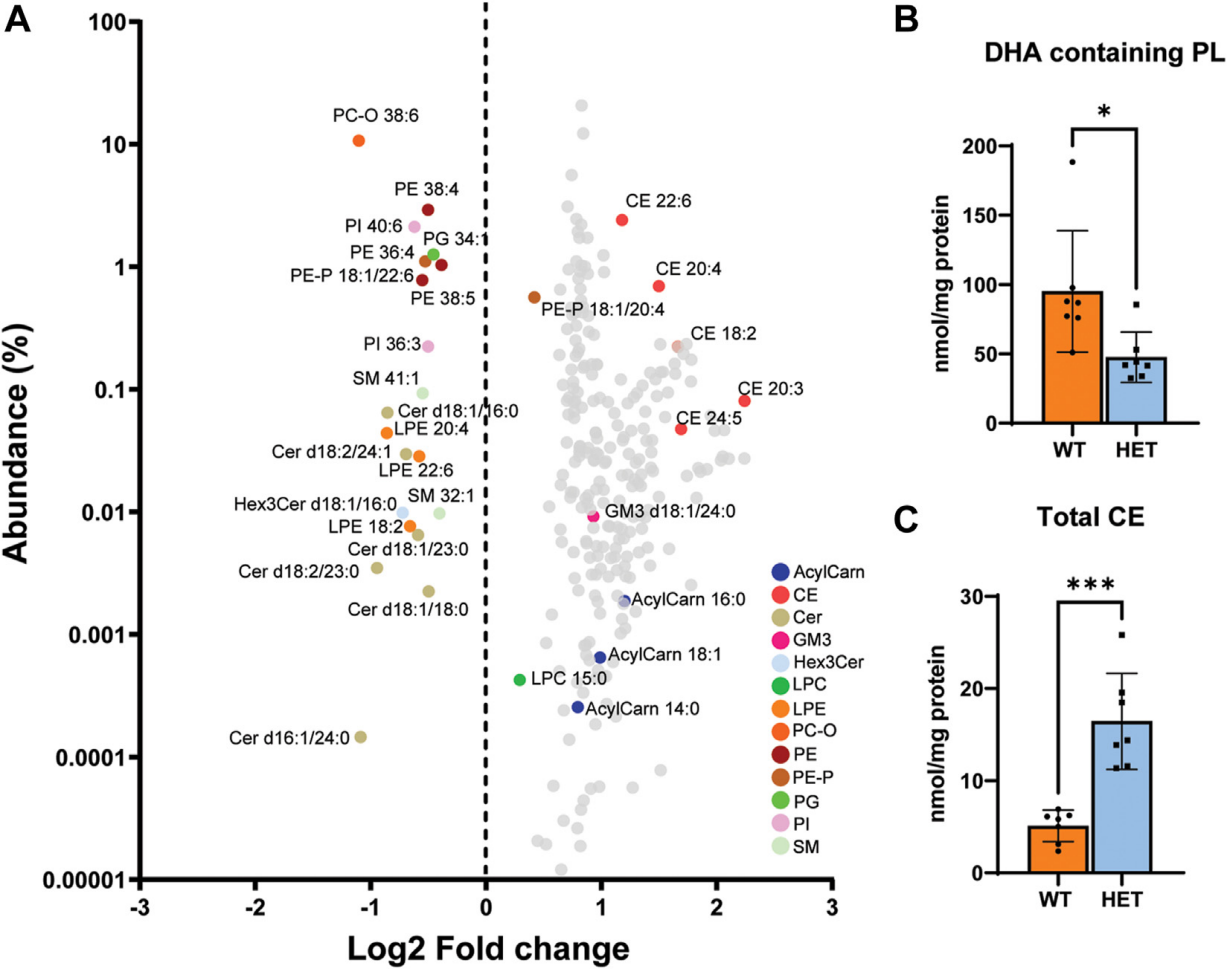
HET kidneys have lower levels of DHA-containing phospholipids and higher levels of cholesteryl esters. A: Lipidomic analysis of WT and HET kidney tissues at day 10 post-IRI. Lipid species plotted as a function of abundance versus fold change. Only statistically significant changed lipid species are indicated by colored dots. B: Total DHA containing phospholipids and, (C) total cholesteryl esters (CE) are plotted on the right (n = 7 per genotype). Data are presented as mean ± SD. *P* < 0.001 ∗∗∗, *P* < 0.01 ∗∗, *P* < 0.05 ∗.

Since Mfsd2a is an LPC transporter with high specificity for LPC containing DHA and that HET mice showed poor renal function and recovery post-IRI, we hypothesized that Mfsd2a in the kidney is critical during the recovery phase by contributing to phospholipid homeostasis. To explore this hypothesis, we performed a targeted lipidomic analysis quantifying 266 lipid species in HET and WT kidneys at day 10 post-IRI. Prior to IRI, lipidomic analysis indicated that HET kidneys had decreases in triglycerides and increases in LPC lipid species, but without significant changes in phospholipid DHA pools relative to WT controls (supplemental Fig. S7). There were limited changes identified for some triglyceride and LPC species but these were of extremely low abundance (<0.1% of total lipids analyzed) and the relevance of changes in these particular lipid species in HET kidneys is not known. Nonetheless, renal function and histology were normal in HET mice at baseline (Fig. 2B and supplemental Fig. S4). From the analysis at day 10 post-IRI, we identified specific DHA containing phospholipids and total phospholipid DHA to be significantly reduced in HET relative to WT kidneys. Moreover, cholesteryl ester species were significantly increased in HET relative to WT kidneys (Fig. 4), which was consistent with increased amounts of cells staining positive for the neutral lipid stain BODIPY493/503, and with many stained cells appearing to be cellular casts (supplemental Fig. S8). These findings indicate that Mfsd2a is critical for maintaining DHA phospholipids following recovery from IRI.

### LPC-DHA treatment ameliorates renal dysfunction after ischemic insult

The DHA deficiency in HET mice following IRI suggests that Mfsd2a is limiting for maintaining kidney DHA levels during the recovery phase after injury. We next explored the concept that plasma LPC-DHA levels are additionally limiting during the recovery phase. Having one functional allele of Mfsd2a in HET mice affords the opportunity to test the hypothesis that treatment with LPC-DHA could ameliorate renal dysfunction in HET mice.

**Fig. 5:**
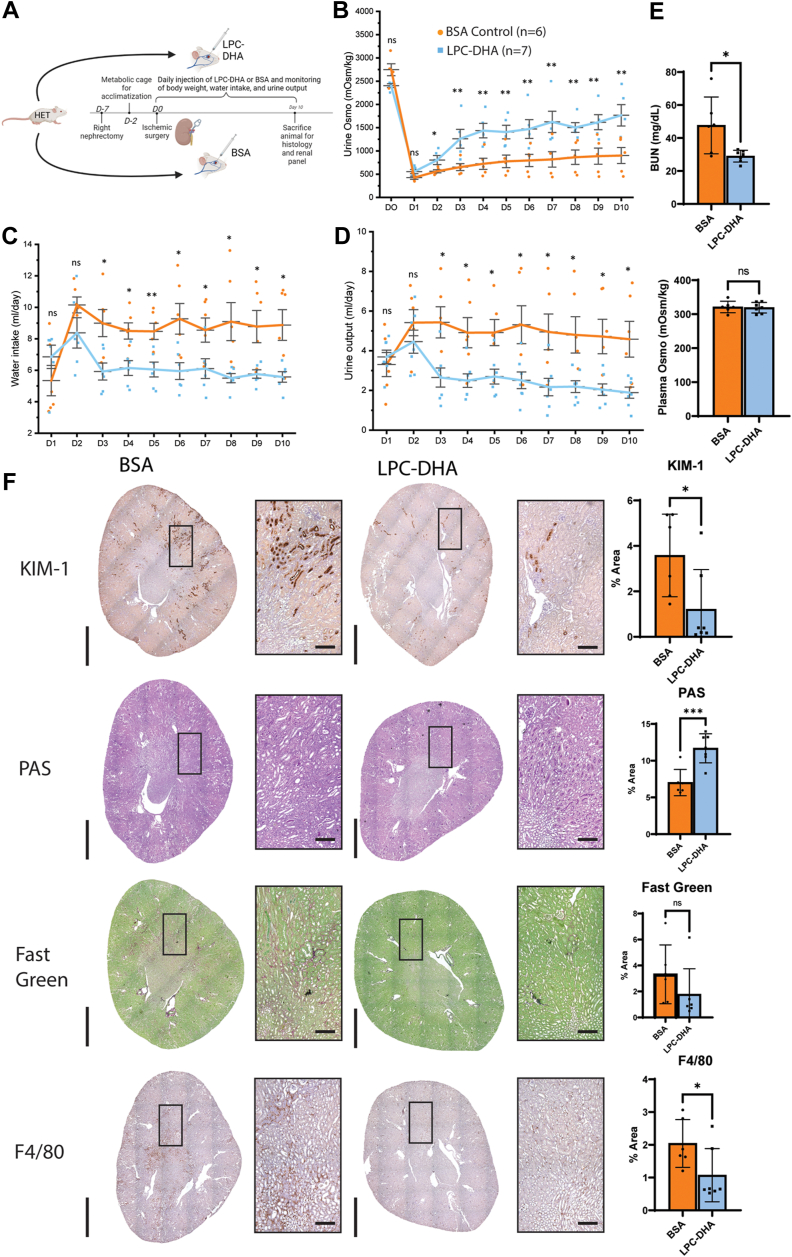
LPC-DHA treatment partially rescued renal function after ischemic insult. A: An experimental timeline of IRI surgery with LPC-DHA treatment in 2a-HET mice. BSA-injected HET mice served as control group. B: Quantification of urine osmolality, (C) urine output, and (D) water intake per day. E: Quantification of BUN and plasma osmolality values at day 10. Data are represented as mean ± SD. F: Histological staining of indicated markers of kidney injury (KIM-1), fibrosis (fast green), and inflammation (F4/80) in LPC-DHA treatment and BSA control groups. Representative images show. Marker levels were quantified as % area of total kidney area and represented as mean ± SD. n = 7 LPC-DHA treated, n = 6 BSA control mice. Scale bar = 3 mm and 100 μm, respectively. *P* < 0.001 ∗∗∗, *P* < 0.01 ∗∗, *P* < 0.05 ∗.

**Fig. 6:**
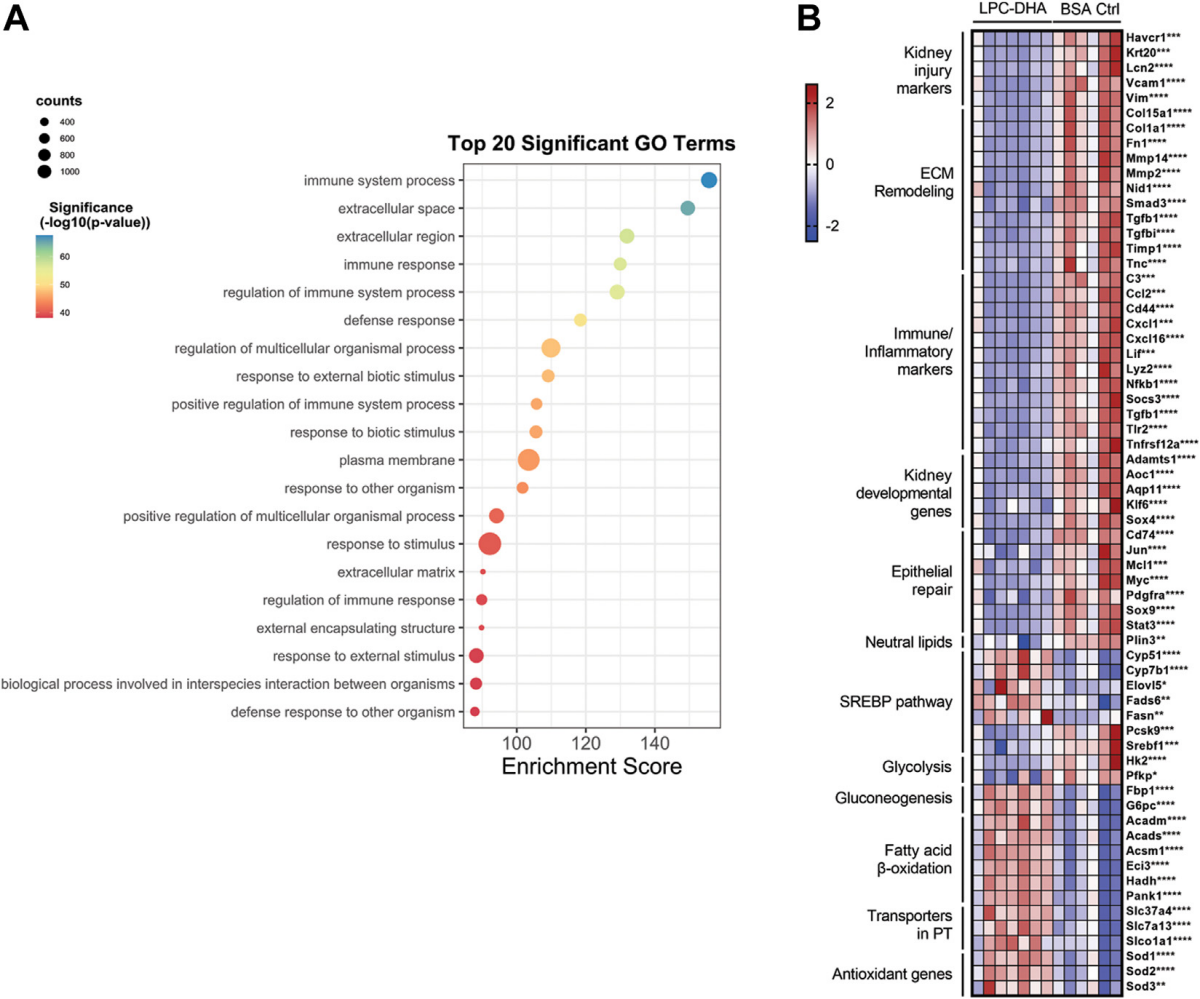
Transcriptomic changes indicative of improved renal recovery from LPC-DHA treatment. A: transcriptomic analysis of kidney tissues in LPC-DHA treated and BSA control groups 10 days after surgery. Shown are the top 20 GO pathways downregulated with LPC-DHA treatment relative to BSA control. B: heatmap of key genes in relevant pathways that are significantly different between LPC-DHA treatment and BSA control groups. n = 7 LPC-DHA treated, n = 6 BSA control mice. *P* < 0.0001 ∗∗∗∗, *P* < 0.001 ∗∗∗, *P* < 0.01 ∗∗, *P* < 0.05∗.

**Fig. 7:**
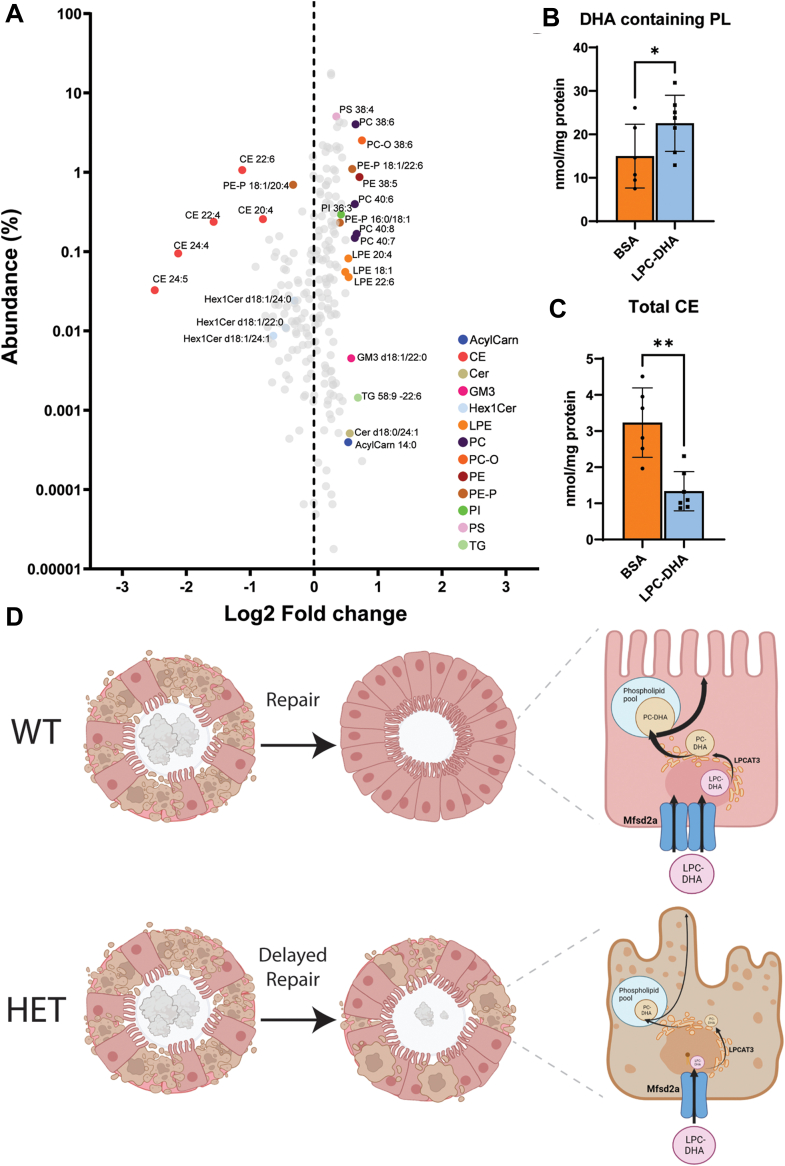
LPC-DHA treatment restores phospholipid DHA levels in HET kidneys. A: lipidomic analysis of BSA control and LPC-DHA treated kidney tissues at day 10 post-IRI. Lipid species plotted as abundance of lipid versus Log_2_ fold change. Only statistically significant changed lipid species are indicated by colored dots. B: total DHA containing phospholipids and, (C) total cholesteryl esters (CE) plotted on the right n = 7 LPC-DHA treated, n = 6 BSA control mice. Data are represented as mean ± S.D. *P* < 0.01 ∗∗, *P* < 0.05 ∗. D: proposed mechanism for the role of Mfsd2a in AKI. Brush border membranes of the S3 segment are sloughed off as debris during renal ischemia. These membranes are rich in phospholipids such as PC, sphingomyelin, and PE. Mfsd2a is expressed at the basolateral side of S3 tubules, which transports LPC-DHA and other plasma-derived lysophospholipids to reconstitute the lost membrane lipids following ischemic injury. Reduction of Mfsd2a levels in the HET animals impede renal recovery which is rescued by LPC-DHA supplementation.

For this experimental approach, HET mice were assigned to two groups. One group received daily intravenous (i.v) injections of LPC-DHA (200 μg) in complex with BSA, while the control group received the same amount of BSA (Fig. 5A). Remarkably, LPC-DHA treatment was able to significantly restore the kidney’s ability to concentrate urine, with higher urine osmolality in the treatment group (Fig. 5B). The LPC-DHA treatment group also exhibited significantly reduced polyuria, polydipsia, and plasma BUN without changes in plasma osmolality (Fig. 5C–E). This improved renal function in the LPC-DHA treated group correlated with significant improvements across all histological markers of injury (Fig. 5F). Consistent with these improvements in renal function in the LPC-DHA treated group, RNA-Seq analysis showed reversals in many of the top upregulated and downregulated pathways pertaining to fibrosis, inflammation, and metabolism originally identified in comparisons of HET relative to WT kidneys (Figs. 3 and 6). Finally, lipidomic analysis of kidneys at day 10 post-IRI revealed that the LPC-DHA treated group showed a reversal in lipidomic changes, namely they exhibited significantly increased levels of DHA-containing phospholipids and decreased levels of cholesteryl esters relative to the control group (Fig. 7A–C). Similar to improvements in renal function, there was variation in lipidomic changes in response to treatment. Importantly, we found that the levels of the abundant DHA-containing phospholipids, namely, PC-38:6, PC-O 38:6, and PC-40:6 correlated with improved renal function (supplemental Fig. S10). Taken together, these data support the conclusion that both Mfsd2a and LPC-DHA are limiting for supplying DHA-containing phospholipids to the epithelium of the S3 segment that is important during the repair process following IRI-induced injury.

## Discussion

In this study, we presented experimental evidence that the lysolipid transporter Mfsd2a is critical for the restoration of renal function following acute IRI through its ability to transport LPC into the S3 segment epithelium of the kidney proximal tubule. We demonstrated that Mfsd2a is limiting for recovery of renal function from IRI and that supplementation with LPC-DHA after IRI can ameliorate renal injury and improve recovery in mice haploinsufficient for Mfsd2a. This study represents the first demonstration that plasma-derived phospholipid uptake by proximal tubules is of physiological importance to renal function and recovery from AKI.

The kidney is made up of millions of nephrons, whereby each nephron functions as a unit contributing to overall kidney function. Different parts of the nephron segment are composed of distinct specialized cell types with their corresponding unique set of transporters defining the particular nephron segment. In humans, the PT reabsorbs 70%–80% of the 180 l of glomerular filtrate daily as well as most of the solutes such as sodium, potassium, and bicarbonate. A high density of mitochondria is essential to support the large energy demands needed to drive transporters (e.g. Na/K ATPase, Na–H exchanger, SGLT1/2) at the PT (36).

Membrane lipids have long been known to affect the function of transporters at the plasma membrane (37). For example, cholesterol loading has been reported to modulate the activity of the Na/K ATPase at basolateral membranes of bovine kidneys and organic cationic transporters in rat brush border membranes (38, 39). However, there is still much to be understood regarding the lipid composition along the PT and whether lipid composition is unique to each segment of the PT. Mfsd2a is a lysolipid transporter with a substrate preference for lysolipids carrying polyunsaturated fatty acids (PUFA) (14). Furthermore, we demonstrated that Mfsd2a is specifically expressed in S3, suggesting a specific requirement for LPC-PUFA uptake in regulating the function of the S3 segment. Spatial lipidomic analysis using mass spectrometry imaging (MSI) has shown that the S3 segment (outer medulla) is enriched in numerous phospholipids species including phosphatidylserine, phosphatidylinositol, and phosphatidylglycerol that contain omega-3 PUFAs such as DHA (40, 41).

The S3 in particular has a thick brush border membrane to facilitate its reabsorption functions. The S3 segment sits deep within the renal cortex, at the corticomedullary junction, and is poorly vascularized. Given these anatomical characteristics of the S3 segment, even under normal physiological conditions, the oxygen supply to the S3 segment is considered just adequate enough to meet its metabolic demands, which rely primarily on fatty acid beta-oxidation and mitochondrial oxidative phosphorylation for ATP production to drive solute transport (42). Consequential to IRI, severe ATP depletion ensues while the reperfusion process leads to build up of reactive oxygen species, loss of autoregulation, endothelial dysfunction, and increased renal vasoconstriction leading to tubular damage and apoptosis (42, 43). The combination of high metabolic demands of S3, low oxygen supply, high mitochondrial density, and reliance on oxidative phosphorylation exacerbates the injury process and makes the S3 segment the most vulnerable part of the kidney to IRI injury. Ischemic injury in the S3 segment is commonly characterized by blebbing of brush border membrane vesicles into the luminal space contributing to intraluminal obstruction (5), suggesting that recovery from injury would require both new cell replacement and rebuilding of brush border membranes, processes that require a source of phospholipid.

Phospholipids can be synthesized de novo through the Kennedy pathway. However, PC production by this pathway is energetically costly. Single-cell sequencing data showed that Kennedy pathway enzymes are expressed at very low levels in the S3 segment (13) which might be insufficient to meet the increased demands for phospholipids following IRI. Given the experimental data gathered in the current study, we propose the following model (Fig. 7D): the S3 segment is metabolically stressed under normal physiological conditions and becomes ATP depleted following IRI, uptake of plasma-derived LPCs via Mfsd2a provides a source of phospholipids to maintain phospholipid homeostasis in the S3 segment and one that could become critical for membrane repair following IRI. This concept is analogous to the role Mfsd2a plays in brain development, where the brain outsources part of its phospholipid supply to the liver which generates LPCs rather than rely entirely on de novo synthesis of its full complement of phospholipids (26). Like the S3 segment, neurons are also in an energy-thrifty state using ATP largely for transport functions rather than intermediary metabolism (44).

A recent study by Humphrey’s group using a similar kidney IRI model as we used here identified two types of injured PT epithelium cell states following IRI that either repaired to normal (Type1) or failed to repair (Type2), with the latter cell state associated with fibrosis (45). Single-cell sequencing data analyzed from that study revealed that Mfsd2a expression was enriched in Type1 S3 PT epithelium relative to Type2. Moreover, the expression of lysophosphatidylcholine acyltransferase 3 (*Lpcat3*), a Lands’ cycle enzyme that converts LPC into PC, was also enriched in Type1 S3 PT cells relative to Type2 cells, indicating that both LPC transport by Mfsd2a and likely its conversion to PC by LPCAT3 is important for repair of the S3 segment following IRI.

Our lipidomic data showed a deficiency in DHA-containing phospholipids in HET mice after injury, demonstrating Mfsd2a is limiting for maintaining DHA levels in the kidney during the repair process. In addition, LPC-DHA treatment post-injury in HET mice was able to improve kidney function and restore levels of PC-DHA and PE-DHA in kidneys, indicating that LPC-DHA is also limiting during the repair process. One limitation of our study is that we utilized whole kidneys for lipidomic analysis. Given that Mfsd2a is specific to the S3 segment, it is possible that the lipidomic changes we reported were greatly underestimated.

Whether the Mfsd2a/LPC pathway is important for protecting the kidney in other types of kidney diseases is not known. Examination of an snRNA expression dataset from a diabetic kidney disease study indicated that Mfsd2a was significantly reduced in patients relative to controls (46). A closer look at the molecular mechanisms underlying diabetic nephropathy draws many parallels with AKI. Changes in vasoactive molecules such as angiotensin 2 and nitric oxide (NO) are seen in both disease conditions, which impairs blood flow to tubular cells. IGF-1 and FGF drive proximal tubule hypertrophy and hyperplasia (47), a process seen in both diabetic nephropathy and in the renal repair phase after AKI (48, 49). Although the initial trigger differs between diabetic nephropathy and AKI, there are multiple common pathways in both disease conditions. Given that reduced expression of Mfsd2a in the S3 segment or low plasma LPC-DHA could be susceptibility factors for recovery from renal injuries, it is possible that other renal conditions could benefit from LPC-DHA treatment.

## Data Availability

All RNAseq and lipidomics datasets are contained within Supplemental Data. All other data are contained within the manuscript.

## Supplemental data

This article contains supplemental data.

## Conflict of interest

D. L. S. serves as a scientific advisor to Aker BioMarine. All other authors declare that they have no conflicts of interest with the contents of this article.

## References

[1] Lewington A.J., Cerda J., Mehta R.L. (2013). Raising awareness of acute kidney injury: a global perspective of a silent killer. Kidney Int..

[2] Rewa O., Bagshaw S.M. (2014). Acute kidney injury-epidemiology, outcomes and economics. Nat. Rev. Nephrol..

[3] Yang L., Humphreys B.D., Bonventre J.V. (2011). Pathophysiology of acute kidney injury to chronic kidney disease: maladaptive repair. Contrib. Nephrol..

[4] Venkatachalam M.A., Bernard D.B., Donohoe J.F., Levinsky N.G. (1978). Ischemic damage and repair in the rat proximal tubule: differences among the S1, S2, and S3 segments. Kidney Int..

[5] Solez K., Morel-Maroger L., Sraer J.D. (1979). The morphology of "acute tubular necrosis" in man: analysis of 57 renal biopsies and a comparison with the glycerol model. Medicine (Baltimore).

[6] Harayama T., Riezman H. (2018). Understanding the diversity of membrane lipid composition. Nat. Rev. Mol. Cell Biol..

[7] Martín-Saiz L., Guerrero-Mauvecin J., Martín-Sanchez D., Fresnedo O., Gómez M.J., Carrasco S. (2022). Ferrostatin-1 modulates dysregulated kidney lipids in acute kidney injury. J. Pathol..

[8] Molitoris B.A., Dahl R., Hosford M. (1996). Cellular ATP depletion induces disruption of the spectrin cytoskeletal network. Am. J. Physiol..

[9] Sutton T.A., Molitoris B.A. (1998). Mechanisms of cellular injury in ischemic acute renal failure. Semin. Nephrol..

[10] Gorgas K., Teigler A., Komljenovic D., Just W.W. (2006). The ether lipid-deficient mouse: tracking down plasmalogen functions. Biochim. Biophys. Acta.

[11] Braverman N.E., Moser A.B. (2012). Functions of plasmalogen lipids in health and disease. Biochim. Biophys. Acta.

[12] Hise M.K., Mantulin W.W., Weinman E.J. (1984). Fluidity and composition of brush border and basolateral membranes from rat kidney. Am. J. Physiol..

[13] Ransick A., Lindström N.O., Liu J., Zhu Q., Guo J.J., Alvarado G.F. (2019). Single-cell profiling reveals sex, lineage, and regional diversity in the mouse kidney. Dev. Cell.

[14] Nguyen L.N., Ma D., Shui G., Wong P., Cazenave-Gassiot A., Zhang X. (2014). Mfsd2a is a transporter for the essential omega-3 fatty acid docosahexaenoic acid. Nature.

[15] Wong B.H., Chan J.P., Cazenave-Gassiot A., Poh R.W., Foo J.C., Galam D.L. (2016). Mfsd2a is a transporter for the essential omega-3 fatty acid DHA in eye and important for photoreceptor cell development. J. Biol. Chem..

[16] Cater R.J., Chua G.L., Erramilli S.K., Keener J.E., Choy B.C., Tokarz P. (2021). Structural basis of omega-3 fatty acid transport across the blood-brain barrier. Nature.

[17] Wood C.A.P., Zhang J., Aydin D., Xu Y., Andreone B.J., Langen U.H. (2021). Structure and mechanism of blood-brain-barrier lipid transporter MFSD2A. Nature.

[18] Bergman S., Cater R.J., Plante A., Mancia F., Khelashvili G. (2023). Substrate binding-induced conformational transitions in the omega-3 fatty acid transporter MFSD2A. Nat. Commun..

[19] Martinez-Molledo M., Nji E., Reyes N. (2022). Structural insights into the lysophospholipid brain uptake mechanism and its inhibition by syncytin-2. Nat. Struct. Mol. Biol..

[20] Chua G.T.B., Loke R.Y., He M., Chin C.F., Wong B.H., Kuk C.K. (2023). Mfsd2a utilizes a flippase mechanism to mediate omega-3 fatty acid lysolipid transport. Proc. Natl. Acad. Sci. U. S. A..

[21] Sengottuvel V., Hota M., Oh J., Galam D.L., Wong B.H., Wenk M.R. (2023). Deficiency in the omega-3 lysolipid transporter Mfsd2a leads to aberrant oligodendrocyte lineage development and hypomyelination. J. Clin. Invest..

[22] Alakbarzade V., Hameed A., Quek D.Q., Chioza B.A., Baple E.L., Cazenave-Gassiot A. (2015). A partially inactivating mutation in the sodium-dependent lysophosphatidylcholine transporter MFSD2A causes a non-lethal microcephaly syndrome. Nat. Genet..

[23] Guemez-Gamboa A., Nguyen L.N., Yang H., Zaki M.S., Kara M., Ben-Omran T. (2015). Inactivating mutations in MFSD2A, required for omega-3 fatty acid transport in brain, cause a lethal microcephaly syndrome. Nat. Genet..

[24] Harel T., Quek D.Q.Y., Wong B.H., Cazenave-Gassiot A., Wenk M.R., Fan H. (2018). Homozygous mutation in MFSD2A, encoding a lysolipid transporter for docosahexanoic acid, is associated with microcephaly and hypomyelination. Neurogenetics.

[25] Scala M., Chua G.L., Chin C.F., Alsaif H.S., Borovikov A., Riazuddin S. (2020). Biallelic MFSD2A variants associated with congenital microcephaly, developmental delay, and recognizable neuroimaging features. Eur. J. Hum. Genet..

[26] Chan J.P., Wong B.H., Chin C.F., Galam D.L.A., Foo J.C., Wong L.C. (2018). The lysolipid transporter Mfsd2a regulates lipogenesis in the developing brain. PLoS Biol..

[27] Berger J.H., Charron M.J., Silver D.L. (2012). Major facilitator superfamily domain-containing protein 2a (MFSD2A) has roles in body growth, motor function, and lipid metabolism. PLoS One.

[28] Wong B.H., Mei D., Chua G.L., Galam D.L., Wenk M.R., Torta F. (2022). The lipid transporter Mfsd2a maintains pulmonary surfactant homeostasis. J. Biol. Chem..

[29] Madisen L., Zwingman T.A., Sunkin S.M., Oh S.W., Zariwala H.A., Gu H. (2010). A robust and high-throughput Cre reporting and characterization system for the whole mouse brain. Nat. Neurosci..

[30] Mäe M.A., He L., Nordling S., Vazquez-Liebanas E., Nahar K., Jung B. (2021). Single-cell analysis of blood-brain barrier response to pericyte loss. Circ. Res..

[31] Dominguez J.H., Rothrock J.K., Macias W.L., Price J. (1989). Na+ electrochemical gradient and Na+-Ca2+ exchange in rat proximal tubule. Am. J. Physiol..

[32] Weinstein A.M. (1986). A mathematical model of the rat proximal tubule. Am. J. Physiol..

[33] Chisholm D.R., Tomlinson C.W.E., Zhou G.L., Holden C., Affleck V., Lamb R. (2019). Fluorescent retinoic acid analogues as probes for biochemical and intracellular characterization of retinoid signaling pathways. ACS Chem. Biol..

[34] Vaidya V.S., Ramirez V., Ichimura T., Bobadilla N.A., Bonventre J.V. (2006). Urinary kidney injury molecule-1: a sensitive quantitative biomarker for early detection of kidney tubular injury. Am. J. Physiol. Ren. Physiol..

[35] Chang-Panesso M., Kadyrov F.F., Lalli M., Wu H., Ikeda S., Kefaloyianni E. (2019). FOXM1 drives proximal tubule proliferation during repair from acute ischemic kidney injury. J. Clin. Invest..

[36] Forbes J.M. (2016). Mitochondria-power players in kidney function?. Trends Endocrinol. Metab..

[37] van Meer G., Voelker D.R., Feigenson G.W. (2008). Membrane lipids: where they are and how they behave. Nat. Rev. Mol. Cell Biol..

[38] Yeagle P.L., Young J., Rice D. (1988). Effects of cholesterol on (Na+,K+)-ATPase ATP hydrolyzing activity in bovine kidney. Biochemistry.

[39] Nabekura T., Takano M., Inui K. (1996). Cholesterol modulates organic cation transport activity and lipid fluidity in rat renal brush-border membranes. Biochim. Biophys. Acta.

[40] Wang G., Heijs B., Kostidis S., Mahfouz A., Rietjens R.G.J., Bijkerk R. (2022). Analyzing cell-type-specific dynamics of metabolism in kidney repair. Nat. Metab..

[41] Moreno-Gordaliza E., Esteban-Fernández D., Lázaro A., Aboulmagd S., Humanes B., Tejedor A. (2018). Lipid imaging for visualizing cilastatin amelioration of cisplatin-induced nephrotoxicity. J. Lipid Res..

[42] Chevalier R.L. (2016). The proximal tubule is the primary target of injury and progression of kidney disease: role of the glomerulotubular junction. Am. J. Physiol. Ren. Physiol..

[43] Kwon O., Hong S.M., Ramesh G. (2009). Diminished NO generation by injured endothelium and loss of macula densa nNOS may contribute to sustained acute kidney injury after ischemia-reperfusion. Am. J. Physiol. Ren. Physiol..

[44] Attwell D., Laughlin S.B. (2001). An energy budget for signaling in the grey matter of the brain. J. Cereb. Blood Flow Metab..

[45] Li H., Dixon E.E., Wu H., Humphreys B.D. (2022). Comprehensive single-cell transcriptional profiling defines shared and unique epithelial injury responses during kidney fibrosis. Cell Metab..

[46] Wilson P.C., Muto Y., Wu H., Karihaloo A., Waikar S.S., Humphreys B.D. (2022). Multimodal single cell sequencing implicates chromatin accessibility and genetic background in diabetic kidney disease progression. Nat. Commun..

[47] Vallon V. (2011). The proximal tubule in the pathophysiology of the diabetic kidney. Am. J. Physiol. Regul. Integr. Comp. Physiol..

[48] Andersson G., Jennische E. (1988). IGF-I immunoreactivity is expressed by regenerating renal tubular cells after ischaemic injury in the rat. Acta Physiol. Scand..

[49] Zhang G.H., Ichimura T., Wallin A., Kan M., Stevens J.L. (1991). Regulation of rat proximal tubule epithelial cell growth by fibroblast growth factors, insulin-like growth factor-1 and transforming growth factor-beta, and analysis of fibroblast growth factors in rat kidney. J. Cell Physiol..

