# Supplementary material for: Mfsd2a-mediated lysolipid transport is important for renal recovery after acute kidney injury
Source: J Lipid Res. 2023 Jul 17;64(8):100416. doi: 10.1016/j.jlr.2023.100416 (PMC10424216; doi:10.1016/j.jlr.2023.100416)
Supplement: Supplemental Figures [file mmc2.pdf]

Supplemental Figures for

Mfsd2a mediated lysolipid transport is important for renal recovery after acute kidney injury

Randy Y. J. Loke[a], Cheen-Fei Chin[a], Gao Liang[b,c], Bernice H. Wong[a], Dwight L. A. Galam[a], Bryan C. Tan[a], Geok-Lin Chua[a], Shintaro Minegishi[a], Norihiko Morisawa[a], Iulia Sidorov[d,e], Bram Heijs[d,e], Jens Titze[a], Markus R. Wenk[b,c], Federico Torta[b,c], and David L. Silver[a,1]

Author affiliations: [a]Signature Research Program in Cardiovascular and Metabolic Disorders, Duke-National University of Singapore (NUS) Medical School, 169857,Singapore; [b]Singapore Lipidomics Incubator, Life Sciences Institute, NUS, 117456,Singapore; [c]Department of Biochemistry, Yong Loo Lin School of Medicine, NUS,117596, Singapore; [d]Center of Proteomics and Metabolomics, Leiden University Medical Center, Leiden, the Netherlands; [e]The Novo Nordisk Foundation Center for Stem Cell Medicine (reNEW), Leiden University Medical Center, Leiden, the Netherlands.

[1]To whom correspondence may be addressed. Email: david.silver@duke-nus.edu.sg

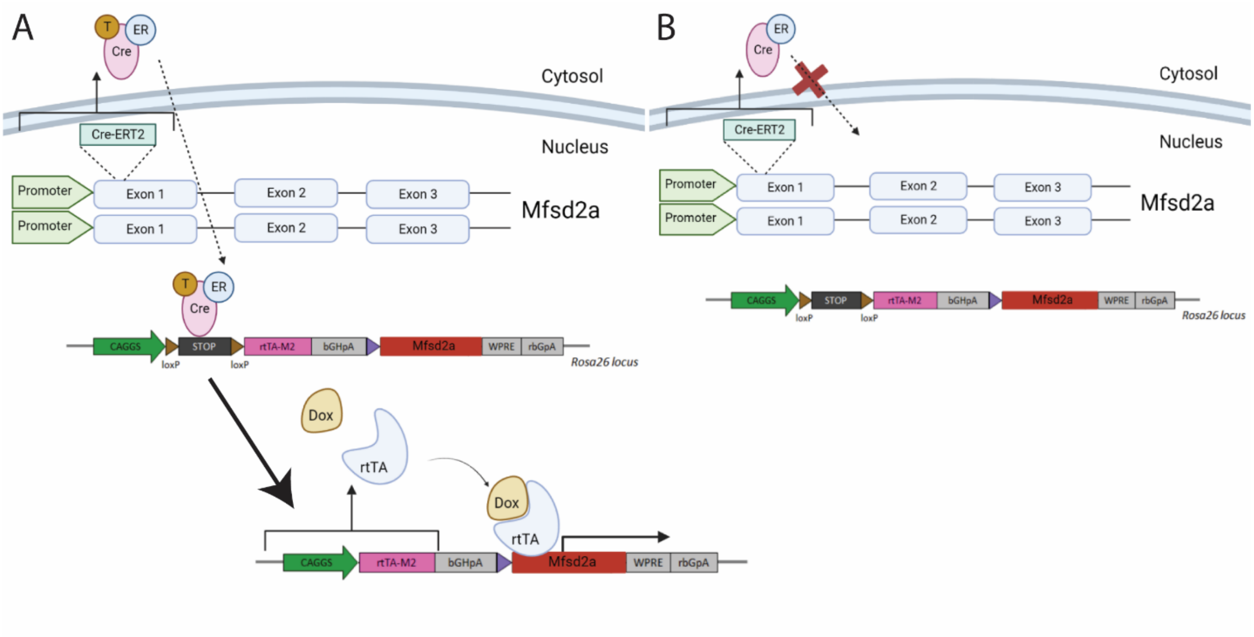

Fig S1. Schematic of the iMfsd2a transgene system. A) Through homologous recombination the human Msfd2a cDNA was inserted into the Rosa26 locus under the control of rtTA (reverse tetracycline-controlled transactivator). rtTA transcription is prevented with a stop codon, flanked by loxP sites. Cre-ER^{T2} (Cre recombinase - estrogen receptor T2) cassette was introduced into exon 1 of the mouse Mfsd2a gene through homologous recombination. Expression of Cre-ER^{T2} is driven by the endogenous Mfsd2a locus. In the presence of Tamoxifen (T), Cre-ER^{T2} enters the nucleus and excises loxP sites at the Rosa26 locus allowing for rtTA expression. rtTA drives expression of Mfsd2a in the presence of doxycycline. B) In the absence of tamoxifen, Cre-ER^{T2} does not enter the nucleus and Mfsd2a at the Rosa26 locus is not transcribed.

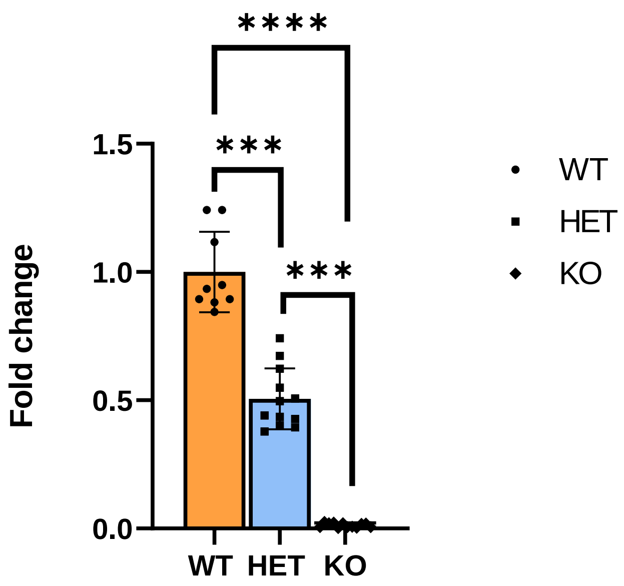

Fig S2. RT-PCR of Mfsd2a transcript levels in Mfsd2a-WT, 2aHET and 2aKO kidneys.
Data are represented as mean ± S.D. n = 9 WT, 12 2aHET, 12 2aKO mice. $p < 0.001$ ***

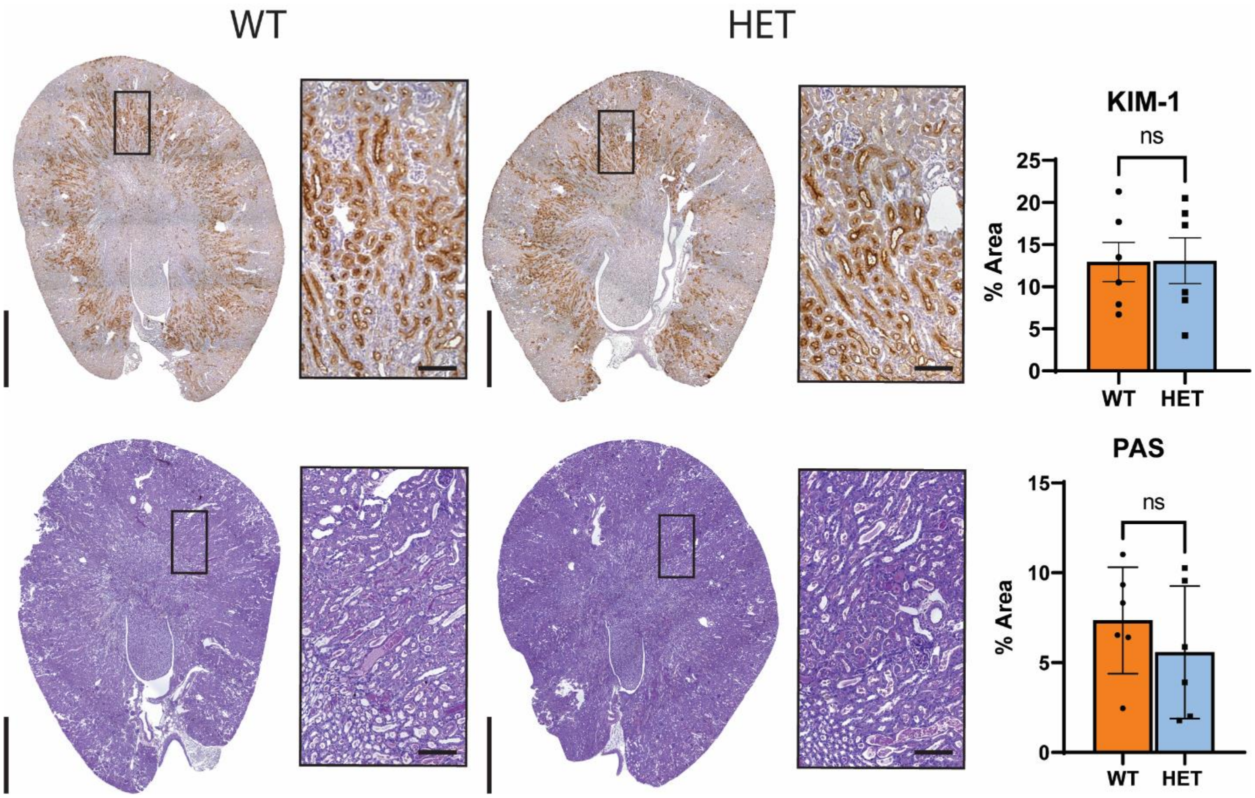

Fig S3. WT and 2aHET mice have similar levels of injury at day 2 post-IRI. Histological staining of KIM-1 and PAS stain between WT and HET animals at day 2 post-IRI. Representative images show. Marker levels were quantified as % area of total kidney area and represented as mean ± S.D. n = 6 WT, 6 2aHET mice. Scale bar = 2mm and 100µm respectively. ns: not significant

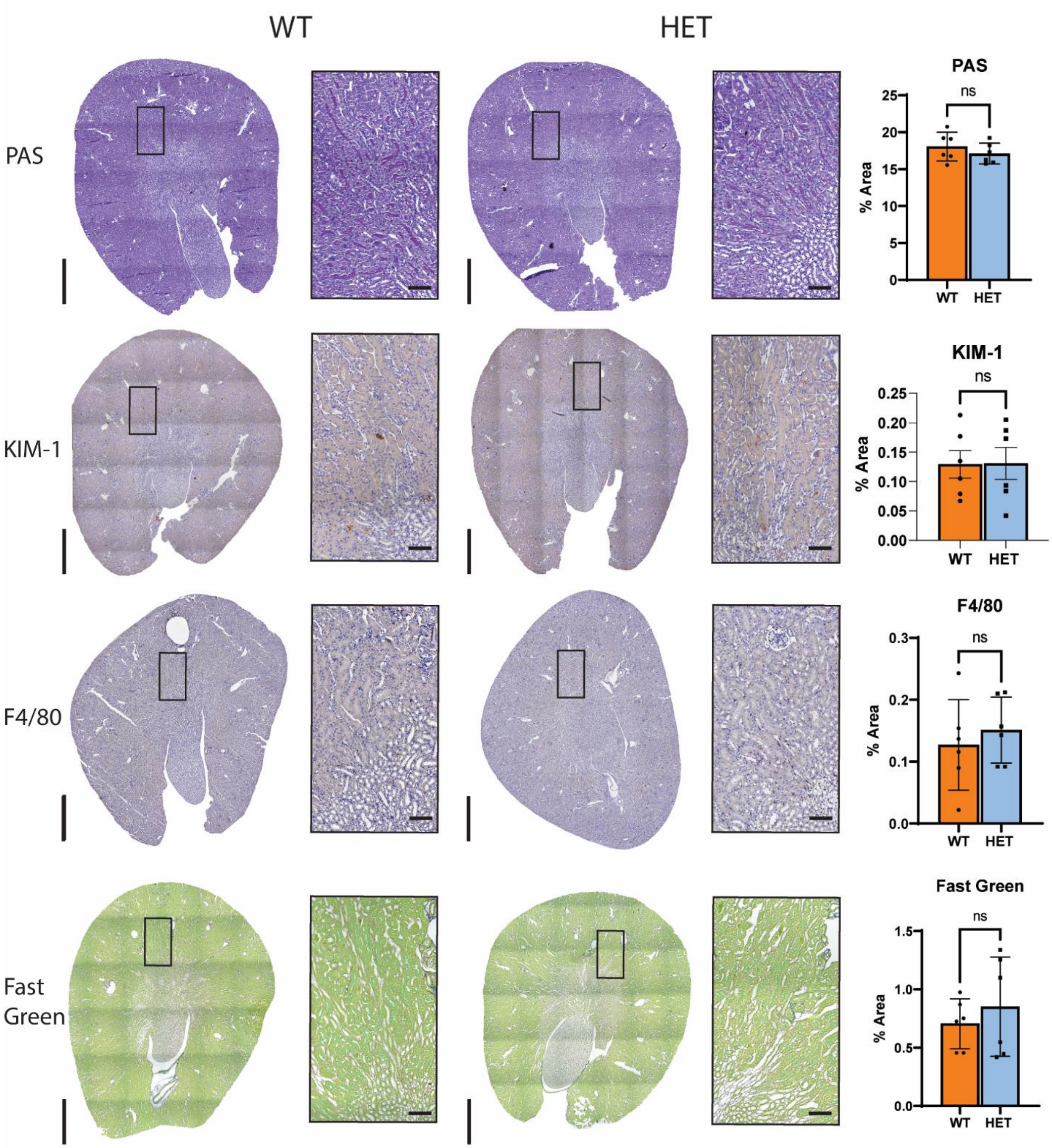

Fig S4. Baseline histology of WT and 2aHET are similar. There was no difference in staining of KIM-1, PAS, acid fast green, and F4/80 between Mfsd2a-WT and Mfsd2a-HET. Marker levels were quantified as % area of total kidney area and represented as mean ± S.D. n = 6 WT, 6 2aHET mice. Scale bar = 2mm and 100μm respectively. ns: not significant

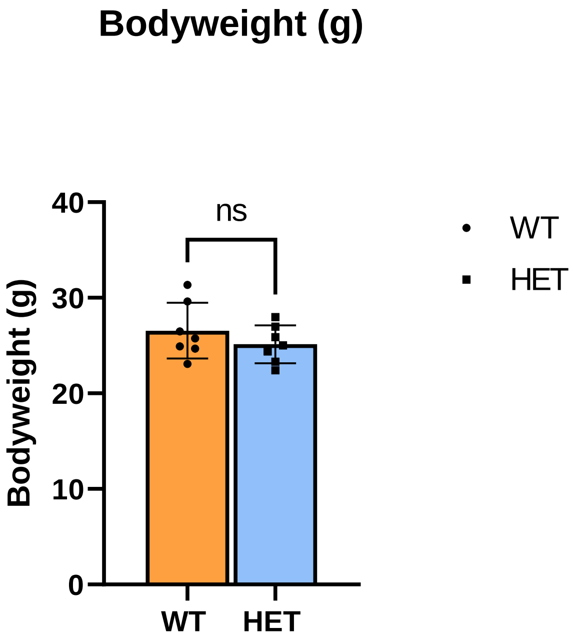

Fig S5. Baseline bodyweight of WT and 2aHET. Data are represented as mean ± S.D. n = 7 WT, 7 2aHET mice. ns: not significant

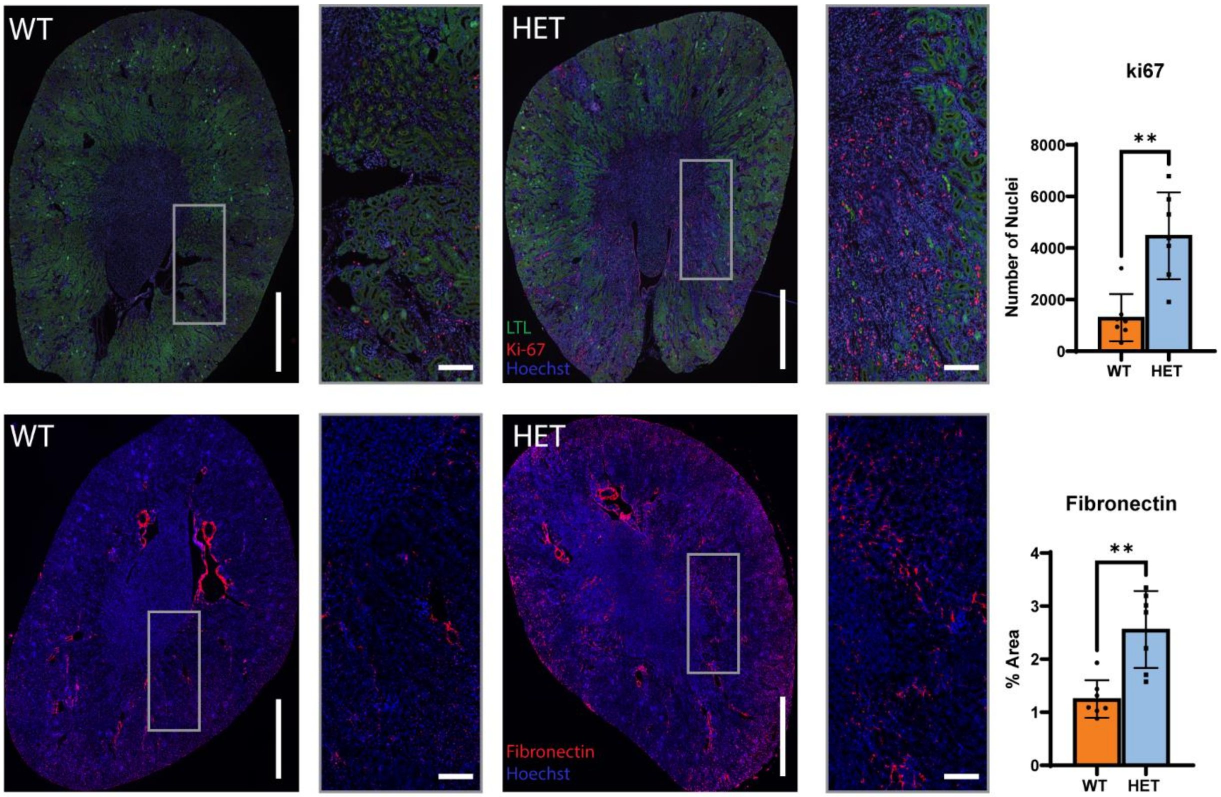

Fig S6. Immunofluorescence staining of Ki-67 and fibronectin in WT and 2aHET mice. Fluorescent area quantified as % area of total kidney area. Data are represented as mean ± S.D. n = 7 WT, 7 HET mice. Scale bar = 2mm and 100µm respectively. $p < 0.01$ **, $p < 0.05$ *

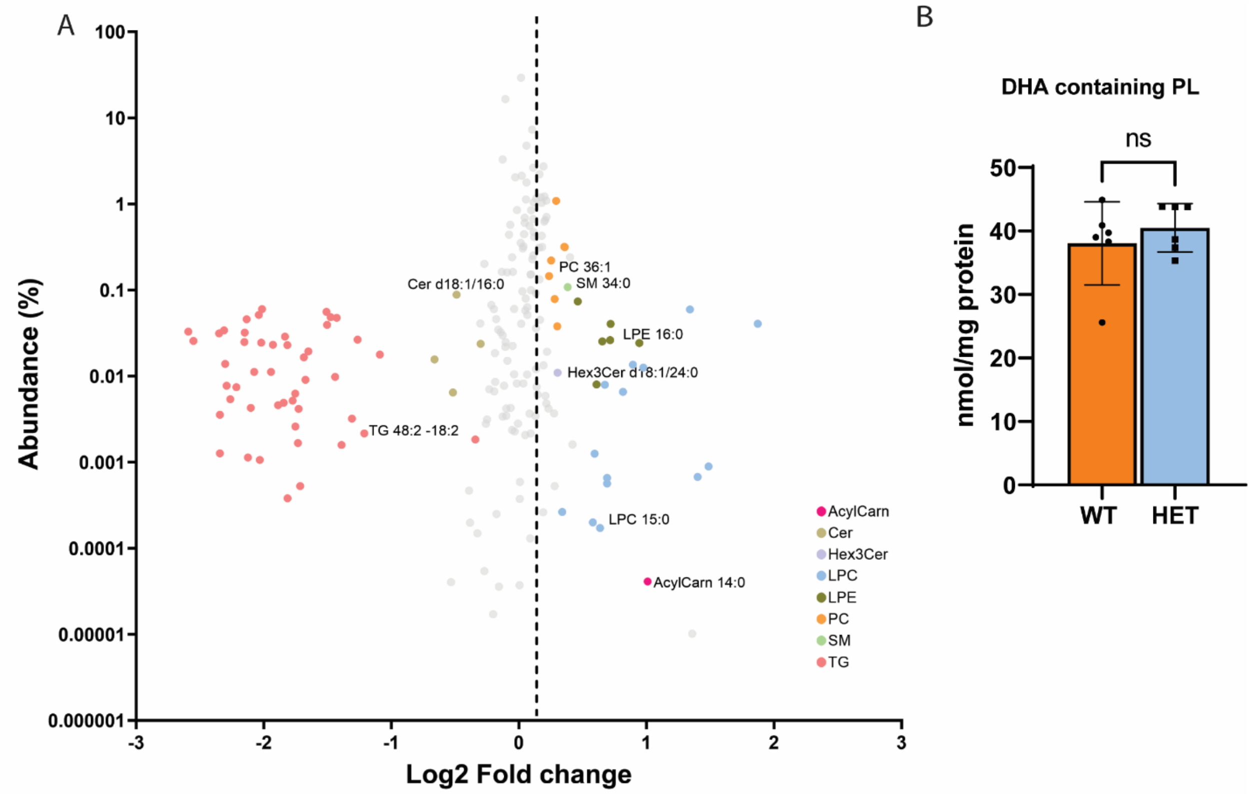

Fig S7. Lipidomic analysis of WT and 2aHET kidneys at baseline A) Lipidomic analysis of WT and 2aHET kidney without injury (baseline) plotted as a function of abundance vs fold change. Only statistically significant changed lipid species are indicated by colored dots. B) Quantification of total DHA containing phospholipids showed no difference between WT and HET kidneys. Data are represented as mean ± S.D. n = 7 WT, 7 2aHET mice. ns: not significant

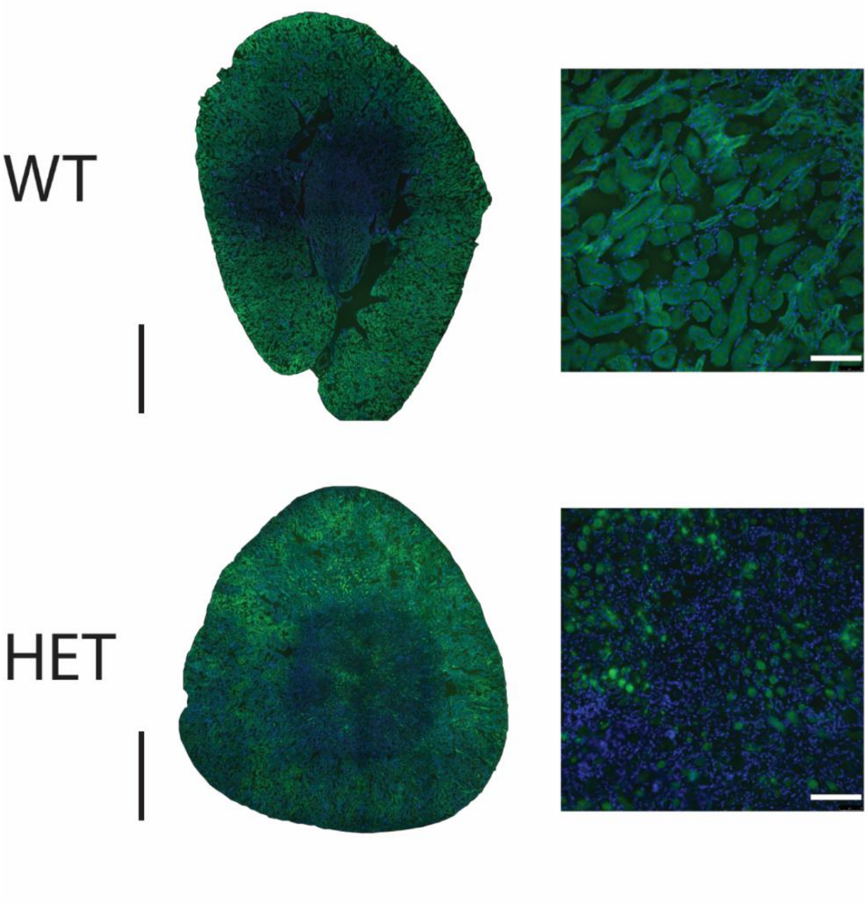

Fig S8. Neutral lipid staing of WT and 2aHET kidneys at day 10 post IRI. Representative images shown. Scale bar = 2mm and 100µm respectively. Kidneys were stained with the neutral lipid fluorescent stain BODIPY493/503. Lefthand side image shows full section of kidney with close-up on the righthand side.

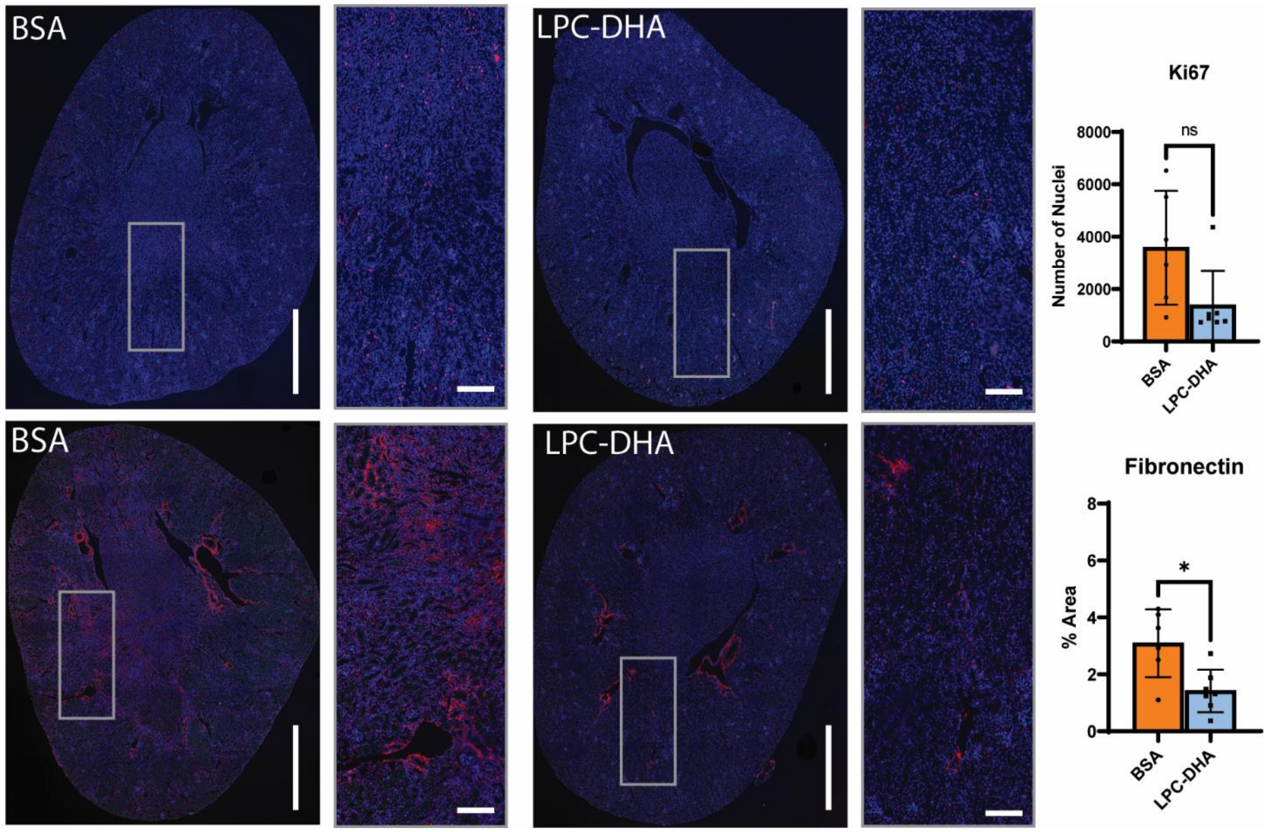

Fig S9. Immunofluorescence staining of Ki-67 and fibronectin in BSA and LPC-DHA treated mice at day 10 post-IRI. Indicated markers were quantified as % area of total kidney area. Marker levels were quantified as number of nuclei (Ki67) or % area of total kidney area (fibronectin) and represented as mean ± S.D. n = 7 LPC-DHA treated, 6 BSA control mice. Scale bar = 2mm and 100µm respectively. $p < 0.01$ **, $p < 0.05$ *

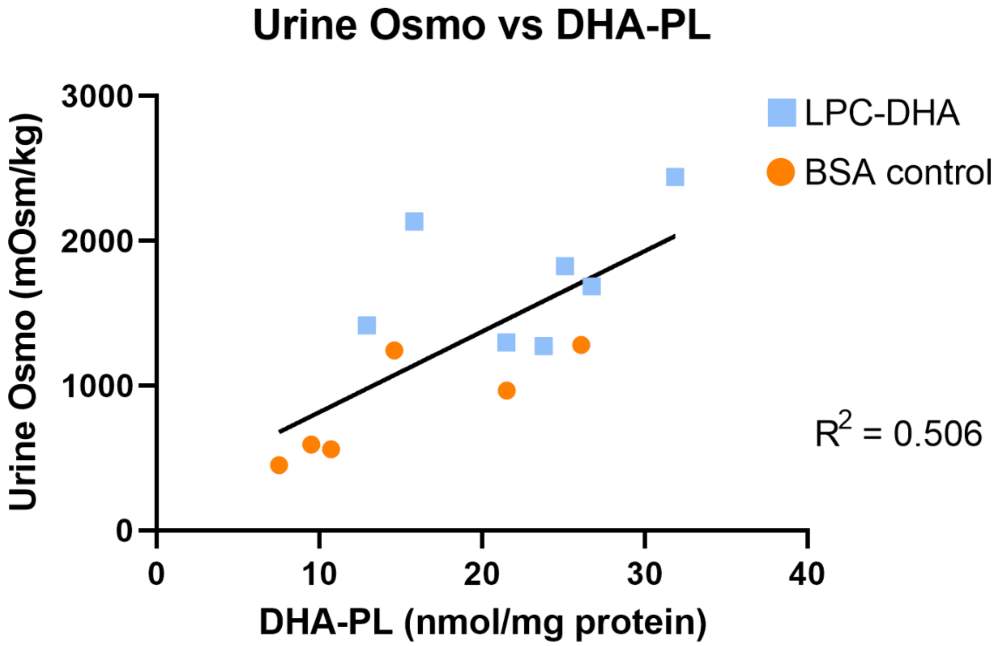

Fig S10. Correlation between urine osmolality and DHA containing phospholipids after treatment. Levels of DHA containing phospholipids (PC-O 38:6, PE-P 18:1/22:6, PC 40:6, PC 38:6) correlate with urine osmolality, a read-out of renal function. n = 7 LPC-DHA treated, 6 BSA control mice.
